# Facile Synthesis of Electroactive and Electrochromic Triptycene Poly(ether-imide)s Containing Triarylamine Units via Oxidative Electro-Coupling

**DOI:** 10.3390/polym9100497

**Published:** 2017-10-10

**Authors:** Sheng-Huei Hsiao, Yu-Chuan Liao

**Affiliations:** Department of Chemical Engineering and Biotechnology, National Taipei University of Technology, No. 1, Sec. 3, Chunghsiao East Rd., Taipei 10608, Taiwan; ycliao92@gmail.com

**Keywords:** triptycene, poly(ether-imide)s, triphenylamine, carbazole, electrochemistry, electropolymerization, electrochromism

## Abstract

Two bisimide compounds, TPA–TPDI and NPC–TPDI, consisting of a triptycene core and two triphenylamine (TPA) or *N*-phenylcarbazole (NPC) end groups were successfully synthesized by the condensation reactions from 1,4-bis(3,4-dicarboxyphenoxy)triptycene dianhydride with 4-aminotriphenylamine and *N*-(4-aminophenyl)carbazole, respectively. These two monomers could polymerize electrochemically via the oxidative coupling reactions of triarylamine units. The electrochemical and spectroelectrochemical properties of the electro-generated triptycene poly(ether-imide)s (TPA–TPPI and NPC–TPPI) were studied. Both polymers have two colored oxidation states, and TPA–TPPI showed better electrochromic performance than NPC–TPPI.

## 1. Introduction

Since the first synthesis of triptycene by Bartlett in 1942 [1], a variety of triptycene derivatives [2,3,4,5,6,7] have been synthesized and investigated because of their unique rigid three-dimensional frameworks. It has been reported that triptycenes can be used in the design of polymers to introduce internal molecular free volume and, thus, produce polymers with lower dielectric constants [8]. Triptycene polyesters has been reported to exhibit increased modulus and ductility due to molecular threading and molecular interlocking [9,10]. Aromatic polyimides incorporating triptycene units exhibited high thermal stability and enhanced solubility [11,12,13]. The free volume imparted by the triptycene units enhances solubility, while retaining the rigidity of a fully aromatic backbone. Additionally, polyimides including a rigid, shape-persistent triptycene moiety ensure a high intrinsic microporosity to allow fast molecular diffusion, leading to high gas permeability [14,15].

Triphenylamine and carbazole derivatives and polymers are well-known for their electro- and photoactive properties; thus, they are widely investigated for various electro-optical applications such as electrophotography, light-emitting diodes, field-effect transistors, solar cells, electrochromic devices, and memory devices [16,17,18,19,20,21,22]. Over the past decade, many triarylamine-based high-performance polymers (typically, aromatic polyamides and polyimides) have been developed as potential electrochromic and memory materials [23,24]. Incorporating bulky, three-dimensional triarylamine units into the backbones of these polymers usually results in good solubility to organic solvents. Thus, amorphous polymeric layers used in the electro-optical devices can be easily prepared by simple solution coating processes. As a class of attractive electrochromic and memory materials, the triarylamine-containing aromatic polyamides and polyimides have combined useful properties such as easily forming stable polarons, high carrier mobility, high thermal stability, and good film-forming capability and mechanical deformability. Additionally, the introduction of bulky, packing-disruptive triarylamine moieties increases the interchain spacing and reduces the packing efficiency thereby increasing the intrinsic microporosity of the polymer films. This ultimate behavior finds application in gas separation membrane technology, where many works have been focused to enhance gas separation performance through the increase in intrinsic free volume achieved by the incorporation of triarylamine units into the polymer chain [25,26,27].

It is well-known that unprotected triphenylamine (TPA) and *N*-substituted carbazoles such as *N*-phenylcarbazole (NPC) undergo electro-oxidative coupling (dimerization) to tetraphenylbenzidine (TPB) and *N*,*N*′-disubstituted 3,3′-bicarbazyls, respectively [28,29]. With appropriately designed monomers, the TPA or carbazole electrochemically oxidative coupling reaction could be employed as an efficient polymerization method to fabricate electroactive polymer films with potential applications in optoelectronic devices [30,31,32,33,34,35,36,37]. In recent years, we have demonstrated that electrochromic polyamide and polyimide films could be facilely synthesized from the amide- or imide-preformed compounds with terminal TPA or NPC groups through oxidative electro-coupling polymerization [38,39]. Compared with conventional chemical methods, electrochemical polymerization has several advantages in polymer synthesis, such as rapid polymerization and direct film formation on the electrode surface. This not only enlarges the scope of candidate polymers, but also omits the procedure of film casting. In addition, prepared by this method thin films have good adhesion and electrical contact to the electrode surface.

In this article we design and synthesize two novel imide ring-preformed monomers, triphenylamine triptycene diimide (TPA–TPDI) and *N*-phenylcarbazole triptycene diimide (NPC–TPDI), featuring a triptycene moiety as an interior core and terminal electroactive TPA and NPC units. We report their electrochemical polymerization on the electrode surface and the electrochromic properties of the obtained polymer films for their use as an active material in construction of the electrochromic devices. For a comparative purpose, two reference compounds M_1_ and M_2_ with *tert*-butyl groups blocked on the reactive sites of their terminal diphenylamino and carbarzolyl units are also prepared and characterized for electrochemical and electrochromic properties.

## 2. Experimental Section

### 2.1. Materials

**Scheme 1:**
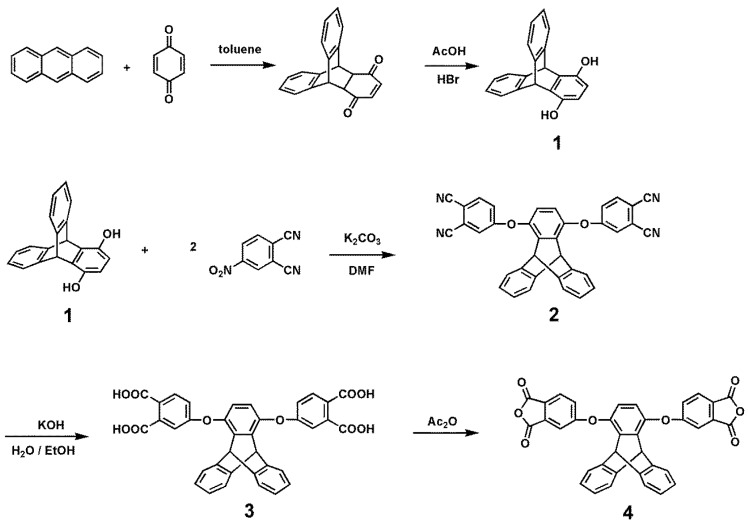
Synthetic route to the triptycene bis(ether anhydride) **4**.

**Scheme 2:**
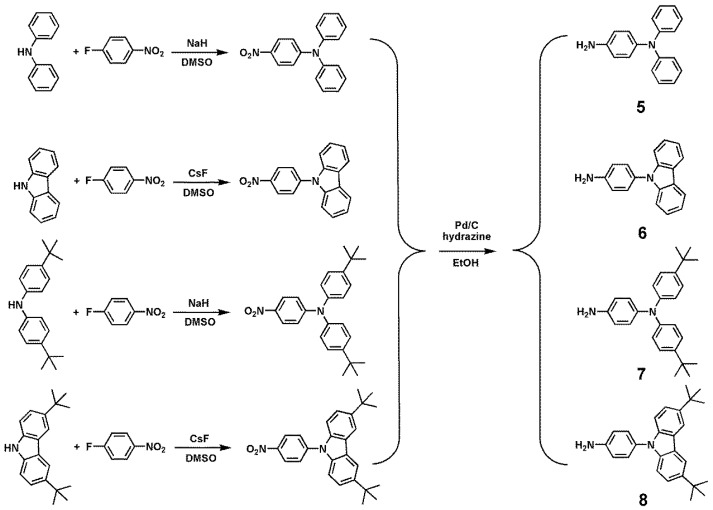
Synthetic routes of the triphenylamine (TPA)- and *N*-phenylcarbazole (NPC)-based amines **5**–**8**.

As reported previously [40], 1,4-bis(3,4-dicarboxyphenoxy)triptycene dianhydride (**4**) was synthesized by the reaction sequence shown in Scheme 1. 4-Aminotriphenylamine (**5**), *N*-(4-aminophenyl)carbazole (**6**), 4,4′-di(*tert*-butyl)-4″-aminotriphenylamine (**7**) and 3,6-di-*tert*-butyl-*N*-(4-aminophenyl)carbazole (**8**) were prepared by the procedures described in literature [41,42] (Scheme 2). Anthracene (Alfa Aesar, Ward Hill, MA, USA), *p*-benzoquinone (Acros, Geel, Belgium), 4-nitrophthalonitrile (Acros), potassium carbonate (K_2_CO_3_, Wako, Osaka, Japan), potassium hydroxide (KOH, Wako), diphenylamine (Acros), carbazole (Acros), *p*-fluoronitrobenzene (Acros), cesium fluoride (CsF, Acros), bis(4-*tert*-butylphenyl)amine (TCI, Tokyo, Japan), 2-chloro-2-methylpropane (*tert*-butyl chloride) (Acros), aluminum chloride (Acros), sodium hydride (Acros), 99% hydrazine monohydrate (TCI), 10% palladium on charcoal (Pd/C, Acros) and acetic anhydride (Ac_2_O, Acros) were used as received from the supplier. *N*,*N*-Dimethylformamide (DMF, Tedia, Fairfield, OH, USA), pyridine (Py, Wako), and *N*,*N*-dimethylacetamide (DMAc, Tedia) were dried over calcium hydride for 24 h, distilled under reduced pressure, and stored over 4 Å molecular sieves in sealed bottles. Tetrabutylammonium perchlorate (Bu_4_NClO_4_, TCI) was recrystallized twice by ethyl acetate under nitrogen atmosphere and then dried in vacuo prior to use. Other reagents and solvents were used as received from commercial sources.

### 2.2. Monomer Synthesis

#### 2.2.1. TPA–TPDI

**Figure 1:**
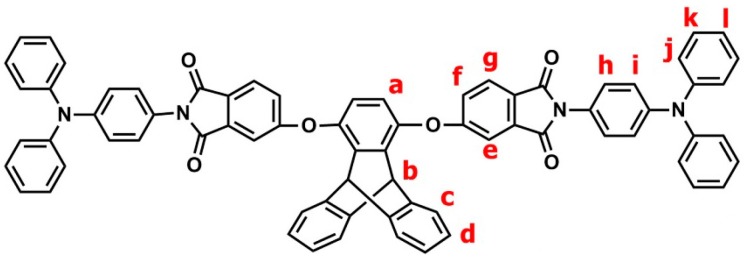
Structure and codes of hydrogen atoms of triphenylamine triptycene diimide (TPA-TPDI).

A dried 50 mL round-bottom flask was charged with 4-aminotriphenylamine (**5**) (1.032 g, 5.0 mmol), triptycene bis(ether anhydride) **4** (1.446 g, 2.5 mmol), and 15 mL of DMAc (15 mL). The mixture was stirred for 10 min at room temperature, after which acetic anhydride (4 mL) and pyridine (2 mL) were added. The reaction mixture was further stirred for 1 h. Then the solution was poured into 150 mL of methanol, a yellow precipitate was obtained by filtration with a 94% yield (2.50 g). IR (KBr): 1786 (asymmetric C=O stretch), 1721 cm^−1^ (symmetric C=O stretch). ^1^H NMR (600 MHz, DMSO-*d*_6_, δ, ppm) (for the peak assignments, see Figure 1): 5.74 (s, 2H, H_b_), 7.01 (dd, *J* = 5.4, 3.2 Hz, 4H, H_c_), 7.02 (s, 2H, H_a_), 7.06−7.11 (m, 16H, H_i,j,l_), 7.33−7.36 (m, 20H, H_d,e,f,h,k_), 7.97 (d, *J* = 8.6 Hz, 2H, H_g_). ESI-MS: 1085.3 [M + Na]^+^; calculated exact mass for C_72_H_46_N_4_O_6_ = 1062.34.

#### 2.2.2. NPC–TPDI

**Figure 2:**
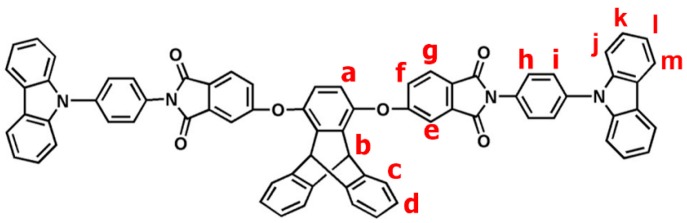
Structure and codes of hydrogen atoms of *N*-phenylcarbazole triptycene diimide (TPA-TPDI).

NPC–TPDI was synthesized in 90% yield from the condensation of *N*-(4-aminophenyl)carbazole (**6**) with triptycene bis(ether anhydride) **4** by a similar synthetic procedure as that of TPA–TPDI. IR (KBr): 1781 (asymmetric C=O stretch), 1716 cm^−1^ (symmetric C=O stretch). ^1^H NMR (600 MHz, CDCl_3_, δ, ppm) (for the peak assignments, see Figure 2): 5.61 (s, 2H, H_b_), 6.86 (s, 2H, H_a_), 7.05 (dd, *J* = 5.5, 3.1 Hz, 4H, H_c_), 7.26−7.28 (m, 6H, H_d_ + H_f_), 7.32 (t, *J* = 7.7 Hz, 4H, H_l_), 7.44 (t, *J* = 8.2 Hz, 4H, H_k_), 7.52 (d, *J* = 8.1 Hz, 4H, H_j_), 7.53 (s, 2H, H_e_), 7.73−7.74 (AB doublets, 8H, H_h_ + H_i_), 7.98 (d, *J* = 8.2 Hz, 2H, H_g_), 8.16 (d, *J* = 7.8 Hz, 4H, H_m_). ESI-MS: 1081.3 [M + Na]^+^; calculated exact mass for C_72_H_42_N_4_O_6_ = 1058.31.

### 2.3. Synthesis of Model Compounds

#### 2.3.1. tBuTPA–TPDI (M1)

**Figure 3:**
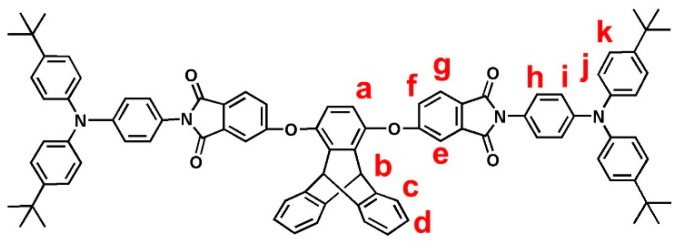
Structure and codes of hydrogen atoms of M1.

By a similar procedure, *t*BuTPA–TPDI (M1) was synthesized as orange powder from 4,4′-di(*tert*-butyl)-4″-aminotriphenylamine (**7**) and triptycene bis(ether anhydride) **4** in 80% yield. IR (KBr): 2956−2861 (*t*-butyl C−H stretch), 1802 (asymmetric C=O stretch), 1728 cm^−1^ (symmetric C=O stretch). ^1^H NMR (600 MHz, DMSO-*d*_6_, δ, ppm) (for the peak assignments, see Figure 3): 1.28 (s, 36H, *t*-butyl), 5.73 (s, 2H, H_b_), 6.95−7.03 (m, 18H, H_a_ + H_c_ + H_i_ + H_j_), 7.26−7.33 (m, 10H, H_d_ + H_f_ + H_h_), 7.34−7.38 (s + d, 10H, H_e_ + H_k_), 7.97 (d, *J* = 7.8 Hz, 2H, H_g_). FAB-MS: 1286.6 (M^+^); calculated exact mass for C_88_H_78_N_4_O_6_ = 1286.59.

#### 2.3.2. tBuNPC–TPDI (M2)

**Figure 4:**
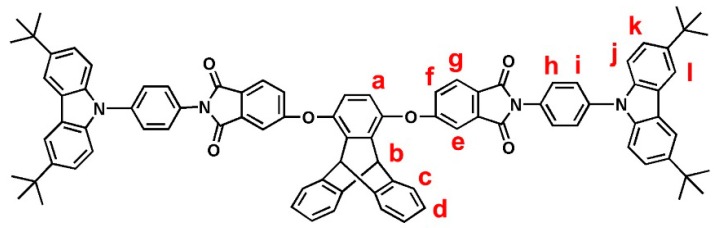
Structure and codes of hydrogen atoms of M2.

Similarly, *t*BuNPC–TPDI (M2) was synthesized from the condensation of 3,6-di-*tert*-butyl-*N*-(4-aminophenyl)carbazole (**8**) with triptycene bis(ether anhydride) **4** in 86% yield. IR (KBr): 2956−2866 (*t*-butyl C−H stretch), 1795 (asymmetric C=O stretch), 1728 cm^−1^ (symmetric C=O stretch). ^1^H NMR (600 MHz, CDCl_3_, δ, ppm) (for the peak assignments, see Figure 4): 1.47 (s, 36H, *t*-butyl), 5.62 (s, 2H, H_b_), 6.86 (s, 2H, H_a_), 7.05 (dd, *J* = 5.5, 3.1 Hz, 4H, H_c_), 7.26−7.27 (m, 6H, H_d_ + H_f_), 7.45 (d, *J* = 8.5 Hz, 4H, H_j_), 7.49 (dd, *J* = 8.5, 1.9 Hz, 4H, H_k_), 7.53 (d, *J* = 2.3 Hz, 2H, H_e_), 7.70 (d, *J* = 8.9 Hz, 4H, H_i_), 7.73 (d, *J* = 8.9 Hz, 4H, H_h_), 7.98 (d, *J* = 8.2 Hz, 2H, H_g_), 8.15 (d, *J* = 1.9 Hz, 4H, H_l_). FAB-MS: 1282.6 (M^+^); calculated exact mass for C_88_H_74_N_4_O_6_ = 1282.56.

### 2.4. Electrochemical Polymerization

Electrochemical polymerization was performed with a CHI 750A electrochemical analyzer. The polymers were synthesized from 2 mM monomers in 0.1 M Bu_4_NClO_4_/dichloromethane (CH_2_Cl_2_) solution by using potentiodynamic method. The polymer was deposited onto the indium tin oxide (ITO)/glass surface with an area of about 0.5 cm × 2.0 cm, and the prepared polymer films were rinsed with acetone to remove the un-reacted monomer and the electrolyte salts.

### 2.5. Fabrication of the Electrochromic Devices (ECDs)

Gel type ECD: Electrochromic polymer films were electrodeposited on the ITO/glass substrate by the electropolymerization method described above. A highly transparent and conductive gel electrolyte based on LiClO_4_ (0.1 g), PMMA (*M*w: 12000) (1 g), and propylene carbonate (1.5 g) was prepared. The gel electrolyte was spread on the polymer-coated side of the electrode. After that, the periphery of the coated polymer film was surrounded with a thermally cured epoxy resin adhesive by a full-auto dispenser, and then a blank ITO glass was covered on the polymer-coated ITO glass to construct the ECD cell. 

Liquid type ECD: A polymer tape with about 30 μm thickness was pasted on the conductive surface of ITO glass. The electrode area frames by the cut out portion is about 2 cm × 2 cm. A solution of 2 mM of compound M1 in 0.1 M Bu_4_NClO_4_/propylene carbonate was filled in the cut out area. Then, the polymer frame was sealed by epoxy resin and another ITO glass was covered on to construct the ECD cell. 

### 2.6. Instrumentation and Measurements

Infrared (IR) spectra were recorded on a Horiba FT-720 FT-IR spectrometer (Kyoto, Japan). ^1^H NMR spectra were measured on a Bruker Avance III HD-600 MHz NMR spectrometer (Rheinstetten, Germany). Tetramethylsilane was used an internal standard. Mass spectra of the synthesized compounds were recorded on a JEOL JMS-700 mass spectrometer (Tokyo, Japan). Agilent 8453 UV–Visible diode-array spectrophotometer (Santa Clara, CA, USA) was used in the UV–Vis absorption and spectroelectrochemical measurements. Cyclic voltammetry and chronoamperometry measurements were performed with a CHI 750A electrochemical analyzer (Austin, TX, USA). The electrochemical cell consists of ITO/glass as a working electrode, a Pt wire as counter electrode, and Ag/AgCl, KCl (sat.) as reference electrode immersed in 0.1 M Bu_4_NClO_4_ in CH_2_Cl_2_ as the supporting electrolyte. Oxidation potentials of the monomers, model compounds, and polymers calibrated to the ferrocene redox couple (+0.44 V vs. Ag/AgCl) were used for the calculation of highest occupied molecular orbital (HOMO)-lowest unoccupied molecular orbital (LUMO) energy levels. The spectroelectrochemical cell was composed of a 1 cm quartz cuvette, ITO/glass as transparent working electrode, a platinum wire as an auxiliary electrode, and a home-made Ag/AgCl, KCl (sat.) reference electrode. Spectroelectrochemical experiments of model compounds were performed with an optically transparent thin-layer electrochemical (OTTLE) cell equipped with an ITO electrode. Absorption spectra in the spectroelectrochemical experiments were measured with an Agilent 8453 UV–Visible diode-array spectrophotometer (Santa Clara, CA, USA). Colorimetric data of the polymer films were measured on an Admesy Brontes colorimeter. Thickness of polymer films was measured with a surface profiler (Kosaka Lab., Surfcorder ET3000, Tokyo, Japan). 

## 3. Results and Discussion

### 3.1. Synthesis of Monomers and Model Compounds

**Scheme 3:**
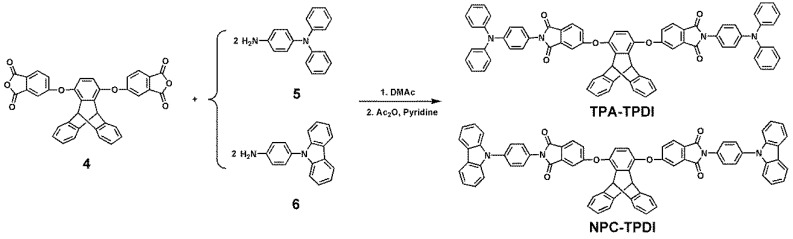
Synthesis of the monomers triphenylamine triptycene diimide (TPA–TPDI) and *N*-phenycarbazole triptycene diimide (NPC–TPDI).

The synthetic route and chemical structures of the monomers TPA–TPDI and NPC–TPDI are shown in Scheme 3. TPA–TPDI and NPC–TPDI were readily prepared by reactions of triptycene bis(ether anhydride) **4** with 4-aminotriphenylamine (**5**) and *N*-(4-aminophenyl)carbazole (**6**), respectively, followed by cyclodehydration with acetic anhydride and pyridine. The structures of the diimide compounds were supported by the FT-IR, ^1^H NMR, and mass spectrometry.

**Figure 5:**
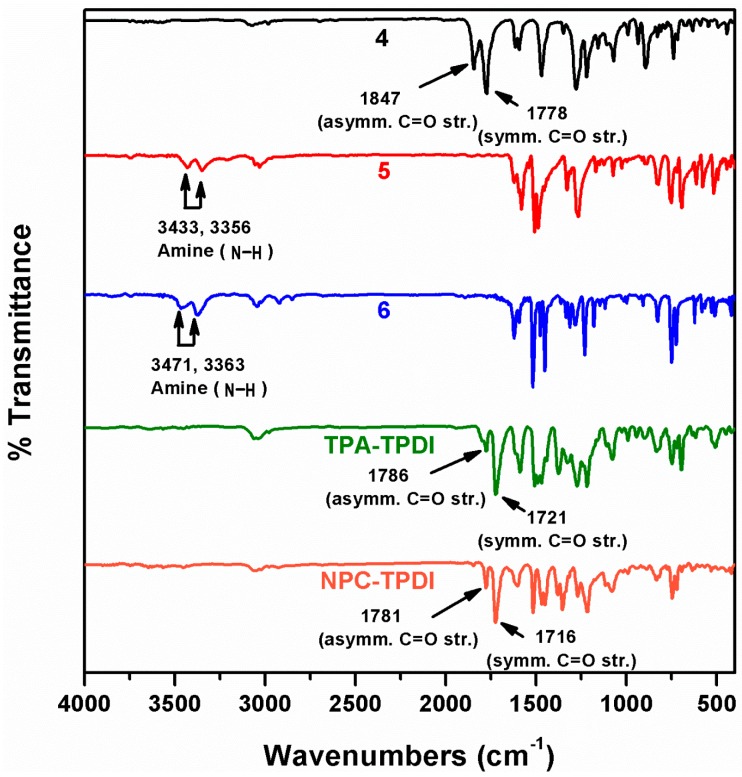
Infrared (IR) spectra of compounds **4**, **5**, **6**, TPA–TPDI and NPC–TPDI.

**Figure 6:**
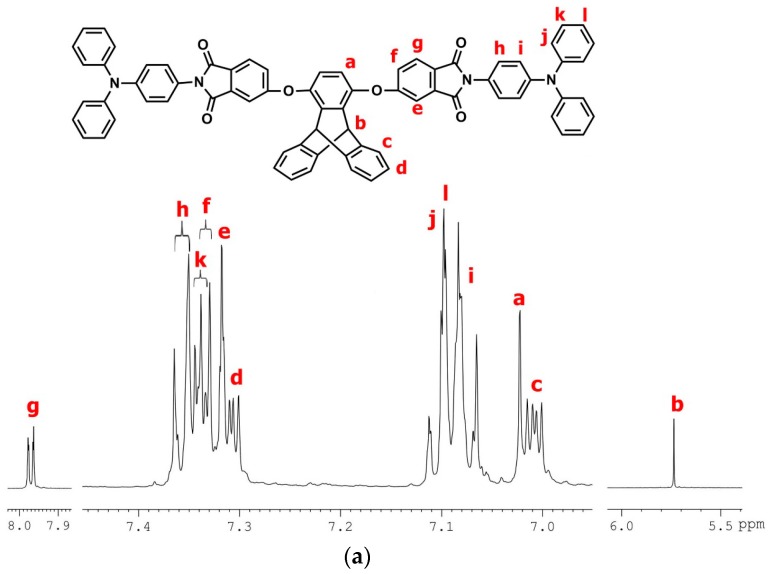

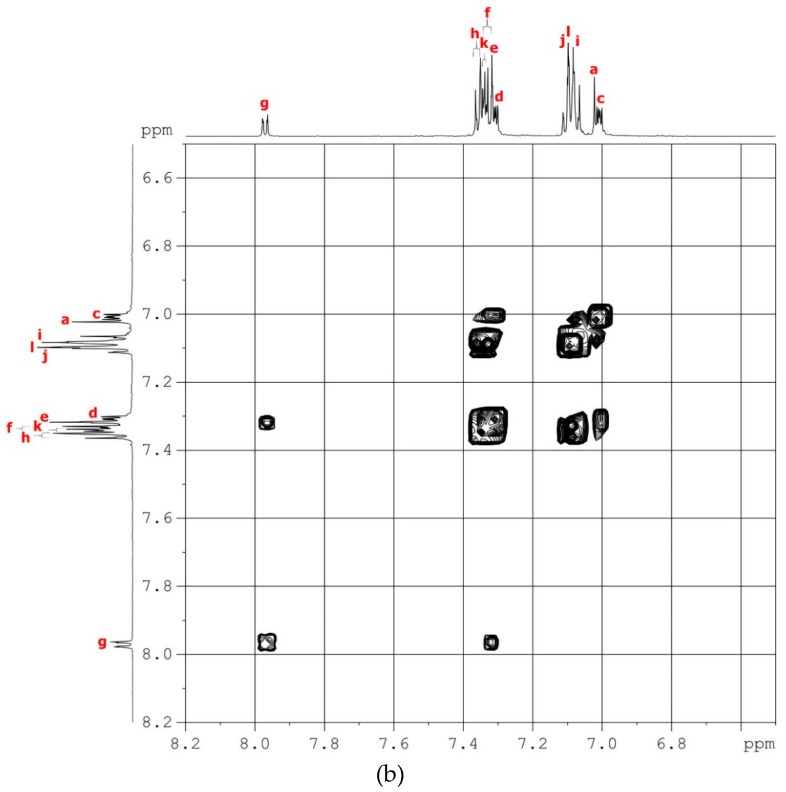
(**a**) ^1^H NMR and (**b**) H–H COSY spectra of TPA–TPDI in DMSO-*d*_6_.

**Figure 7:**
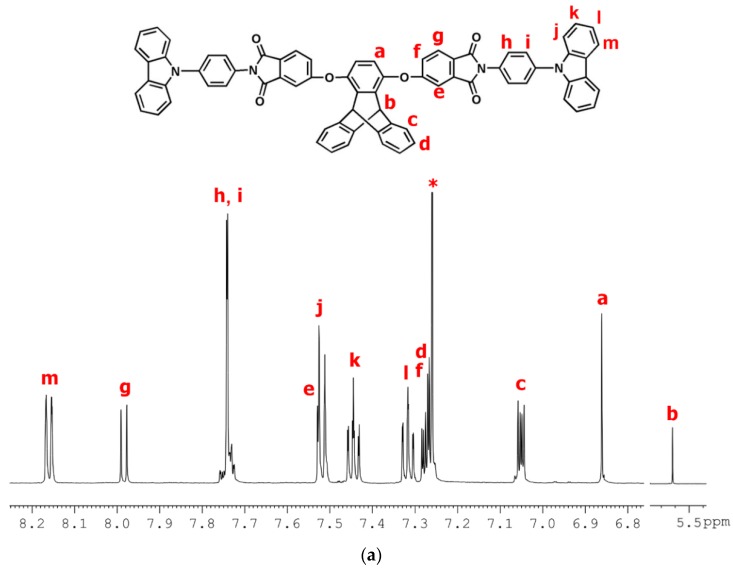

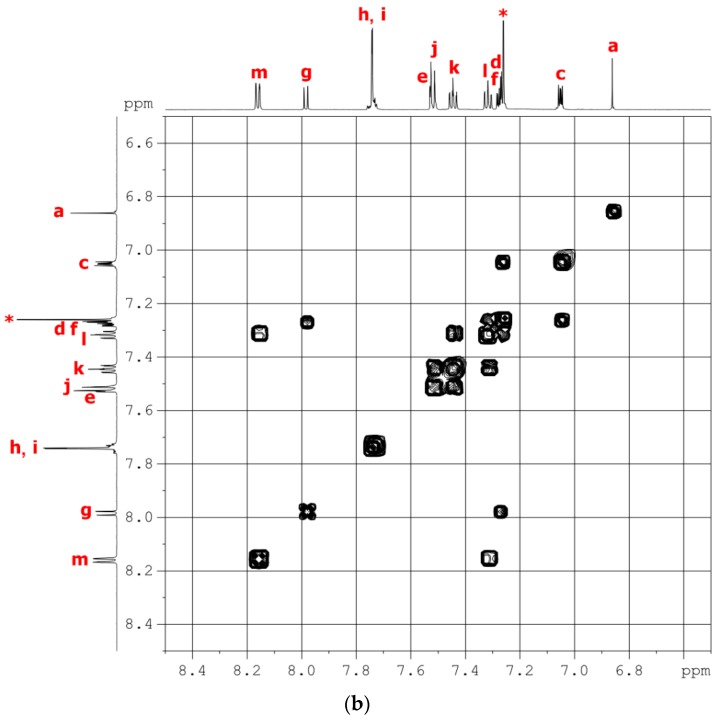
(**a**) ^1^H NMR and (**b**) H–H COSY spectra of NPC–TPDI in CDCl_3_ (* solvent peak).

Figure 5 illustrates FT-IR spectra of the synthesized compounds. The IR spectra of amino compounds **5** and **6** show the typical –NH_2_ stretching absorption pair in the range of 3300−3500 cm^−1^. After condensation with triptycene bis(ether anhydride) **4**, the characteristic absorptions of the amino groups disappeared, and the IR spectra of TPA–TPDI and NPC–TPDI showed the characteristic absorption bands of the imide group at around 1785 and 1722 cm^−1^ (imide asymmetric and symmetric C=O stretching). The ^1^H NMR and H–H COSY spectra of the diimide compounds TPA–TPDI and NPC–TPDI compiled in Figure 6 and Figure 7 agree well with their molecular structures. In addition, mass spectroscopy results of TPA–TPDI and NPC–TPDI are consistent with their calculated values (Supplementary Materials: Figure S4).

**Scheme 4:**
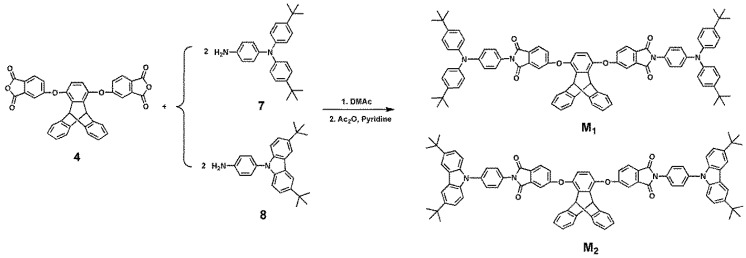
Synthesis of model compounds M1 and M2.

As shown in Scheme 4, two model compounds M1 and M2 with bulky *tert*-butyl groups substituted on the active sites of the terminal diphenylamine or carbazole units were also synthesized from triptycene bis(ether anhydride) **4** with 4,4′-di(*tert*-butyl)-4″-amino-triphenylamine (**7**) and 3,6-di-*tert*-butyl-*N*-(4-aminophenyl)carbazole (**8**), respectively, followed by cyclodehydration with acetic anhydride and pyridine.

Figure S1 (Supplementary Materials) illustrates FT-IR spectra of model compounds M1 and M2 together with compounds **4**, **7**, and **8**. The IR spectra of model compounds M1 and M2 gave characteristic imide absorption bands at around 1795 and 1728 cm^−1^ (imide asymmetric and symmetric C=O stretching) and *tert*-butyl groups showed medium-intensity bands at around 2956−2861 cm^−1^ (sp^3^ C−H stretch).

The ^1^H NMR and H–H COSY spectra of M1 and M2 are presented in Figures S2 and S3 (Supplementary Materials), respectively. The *tert*-butyl hydrogens of M1 and M2 have resonance signals at 1.28 and 1.47 ppm, respectively. Other resonance signals are also in very good agreement with the proposed molecular structures of these two model compounds. Furthermore, mass spectroscopy results of M1 and M2 are consistent with their calculated values (Supplementary Materials: Figures S5 and S6). 

### 3.2. Electrochemical Activity of Monomers and Model Compounds

**Figure 8:**
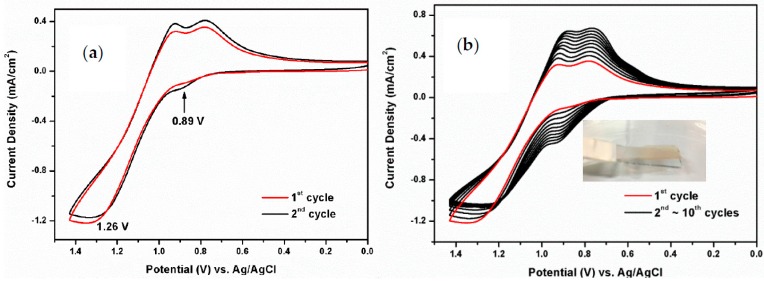
(**a**) The first two cyclic voltammograms and (**b**) ten repeated potential scanning of 2 mM of TPA–TPDI in 0.1 M Bu_4_NClO_4_/CH_2_Cl_2_ solution between 0 and 1.44 V at a scan rate of 50 mV s^−1^.

Multiple oxidation processes were exhibited by the cyclic voltammetry (CV) diagrams of the investigated monomers. They were measured in dichloromethane and the Bu_4_NClO_4_ was used as the supporting electrolyte at a potential scan rate of 50 mV s^−1^. Figure 8 depicts characteristic CV diagrams recorded during sweeping the solution of monomer TPA–TPDI within 0−1.44 V. In the first CV scan, the monomer displayed one oxidation peak at ca. 1.26 V, attributable to the oxidation of TPA. From the first reverse negative potential scan, two cathodic waves at 0.92 and 0.78 V were detected. In the second scan, a new oxidation shoulder appeared at 0.89 V that was the complementary anodic process of the cathodic peak at 0.78 V. This is a typical oxidation wave of the tetraphenylbenzidine (TPB) group, indicating the occurrence of the oxidative coupling between the TPA units. After ten repeated CV scans between 0 and 1.4 V, an electrochemically deposited thin film was observed on the electrode surface (see the inset in Figure 8b). 

**Figure 9:**
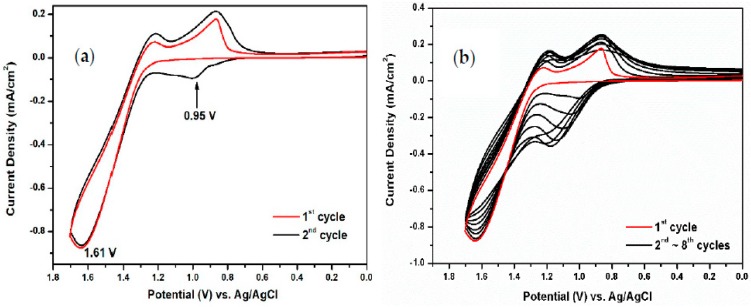
(**a**) The first two cyclic voltammograms and (**b**) eight repeated potential scanning of 2 mM of NPC–TPDI in 0.1 M Bu_4_NClO_4_/CH_2_Cl_2_ solution between 0 and 1.70 V at a scan rate of 50 mV·s^−1^.

**Figure 10:**
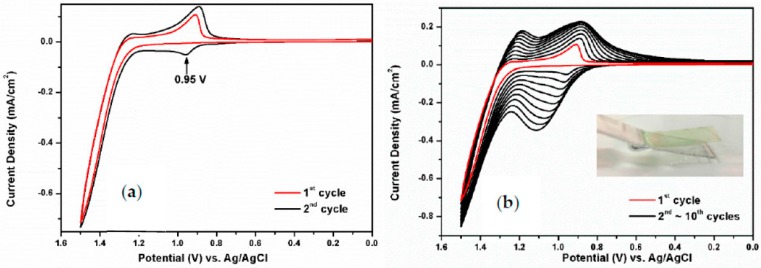
(**a**) The first two cyclic voltammograms and (**b**) ten repeated potential scanning of 2 mM of NPC–TPDI in 0.1 M Bu_4_NClO_4_/CH_2_Cl_2_ solution between 0 and 1.50 V at a scan rate of 50 mV s^−1^.

The repetitive CV diagrams of monomer NPC–TPDI between 0 and 1.70 V are shown in Figure 9. In the first CV scan of NPC–TPDI, an anodic wave peaked at 1.61 V was observed, which is ascribed to the electrochemical oxidation of the terminal carbazole units. However, two waves were found in the backward scan, indicating the formation of biscarbazole units through oxidative coupling of the carbazole groups. In the second CV scan, a new oxidation peak appeared at 0.95 V that corresponded to the first oxidation of the biscarbazole dimer formed in situ during the first CV scan. The relatively high oxidation potential of the carbazole unit might result in an over-oxidation of the compound and undesired side reactions; thus we could not observe stable increasing in the redox couples current densities. Therefore, we slightly lowered the applied voltage; Figure 10 depicts characteristic CV diagrams recorded during sweeping the solution of monomer NPC–TPDI within 0−1.50 V. As the CV sweep continued, a progressive growth in all peak currents was observed. This behavior suggests that the oxidative coupling of the radical cations NPC–TPDI produced a continuous growth of an electroactive and conductive layer on the electrode surface. As shown in the inset in Figure 10b, a robust polymer film can be removed from the ITO-glass surface after immersing in water for a certain period of time.

**Figure 11:**
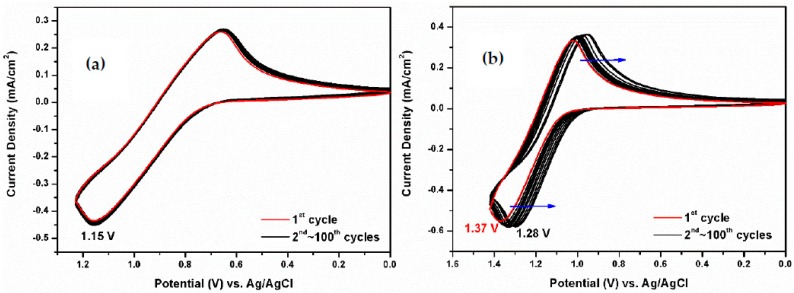
Cyclic voltammetry (CV) diagrams of 2 mM of model compounds (**a**) M1 and (**b**) M2 in 0.1 M Bu_4_NClO_4_/CH_2_Cl_2_ solution at a scan rate of 50 mV·s^−1^.

The CV behaviors of the model compounds M1 and M2 are shown in Figure 11. Model compound M1 showed a reversible redox behavior, with oxidation peak at around 1.15 V. After 100 scans between 0 and 1.23 V, M1 produced almost the same patterns as that observed in the first scan, and no new peaks were detected under these experimental conditions. Model compound M2 also presented a reversible CV behavior, with a slight shift of the anodic and cathodic peak potentials. These two model compounds showed no electrochemical oxidative coupling reaction, and thus no polymer films were built on the electrode surface. This reversible redox behavior could be explained by the blocking of the active sites of the triphenylamine and carbazole units by the bulky *tert*-butyl groups.

**Figure 12:**
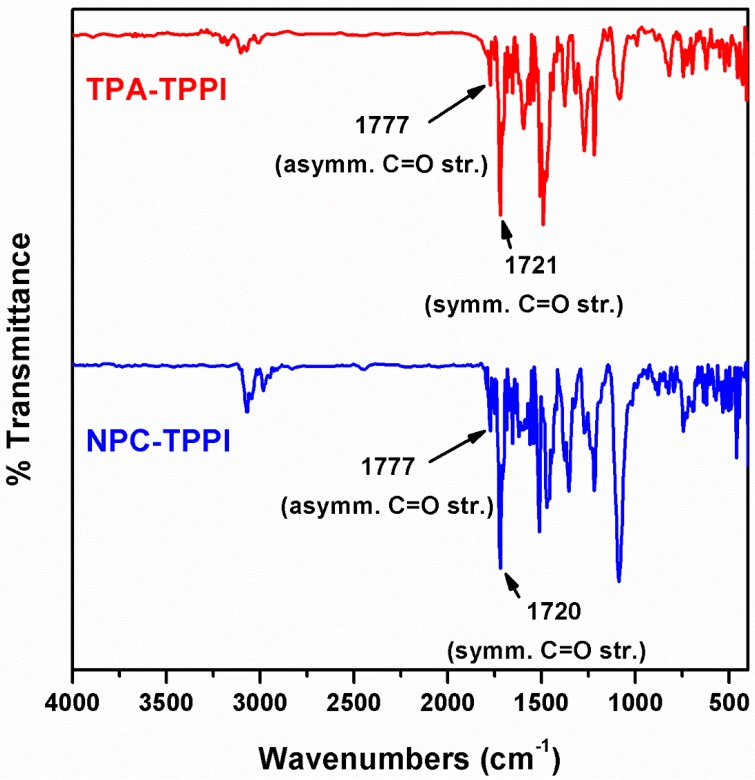
IR spectra of elecytrodeposited polymer films TPA–TPPI and NPC–TPPI.

Figure 12 illustrates FT-IR spectra of electrodeposited polymer films TPA–TPPI and NPC–TPPI. Their IR spectra are similar to those of the precursor monomers. The imide groups of these polymers gave two characteristic bands at around 1777 and 1720 cm^−1^, respectively (imide asymmetric and symmetric C=O stretching).

### 3.3. Optical Properties

**Figure 13:**
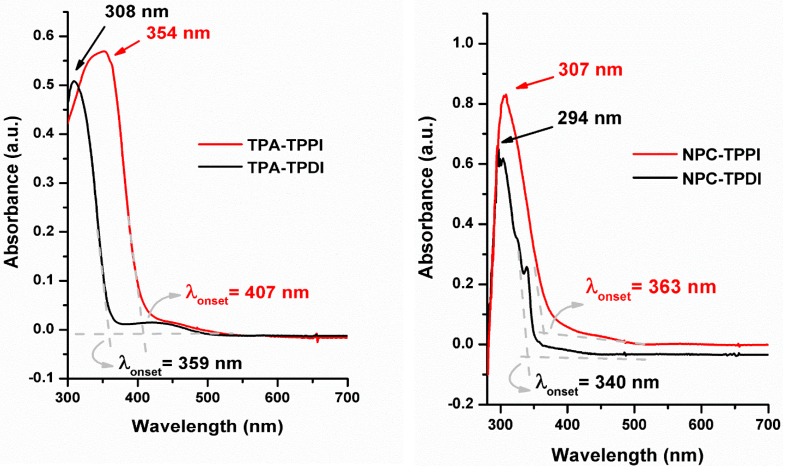
UV–Vis absorption spectra of TPA–TPDI and NPC–TPDI in CH_2_Cl_2_ and the films of TPA–TPPI and NPC–TPPI on ITO-glass.

The UV–Vis absorption spectra of monomer TPA–TPDI and NPC–TPDI in CH_2_Cl_2_ and their deposited polymer films in solid state on an ITO electrode are illustrated in Figure 13. The spectra of the monomers show absorption bands with maximum peaks at 308 and 294 nm, respectively. The polymer films of TPA–TPPI and NPC–TPPI show absorption maxima at 354 and 307 nm, respectively. The slight red-shift of absorption maxima and onsets of the polymer film compared to the monomers implies an extended π-conjugation length.

### 3.4. Redox Response of Polymers

**Figure 14:**
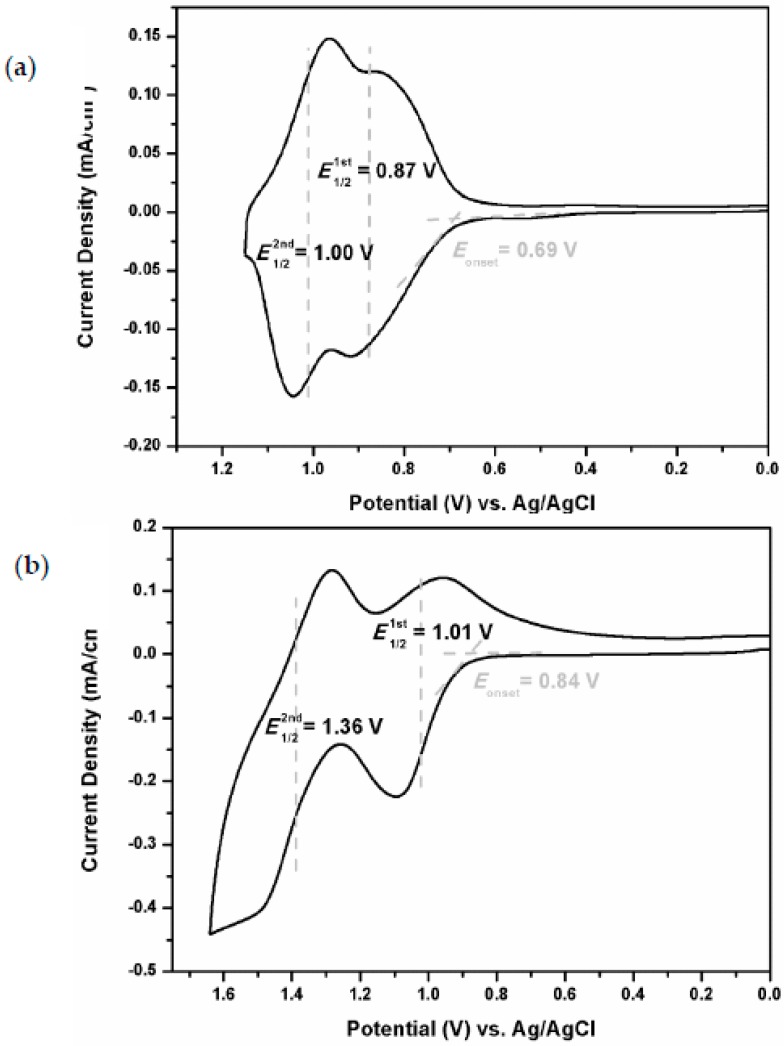
Cyclic voltammograms of (**a**) TPA–TPPI and (**b**) NPC–TPPI films on the ITO-coated glass slide.

**Scheme 5:**
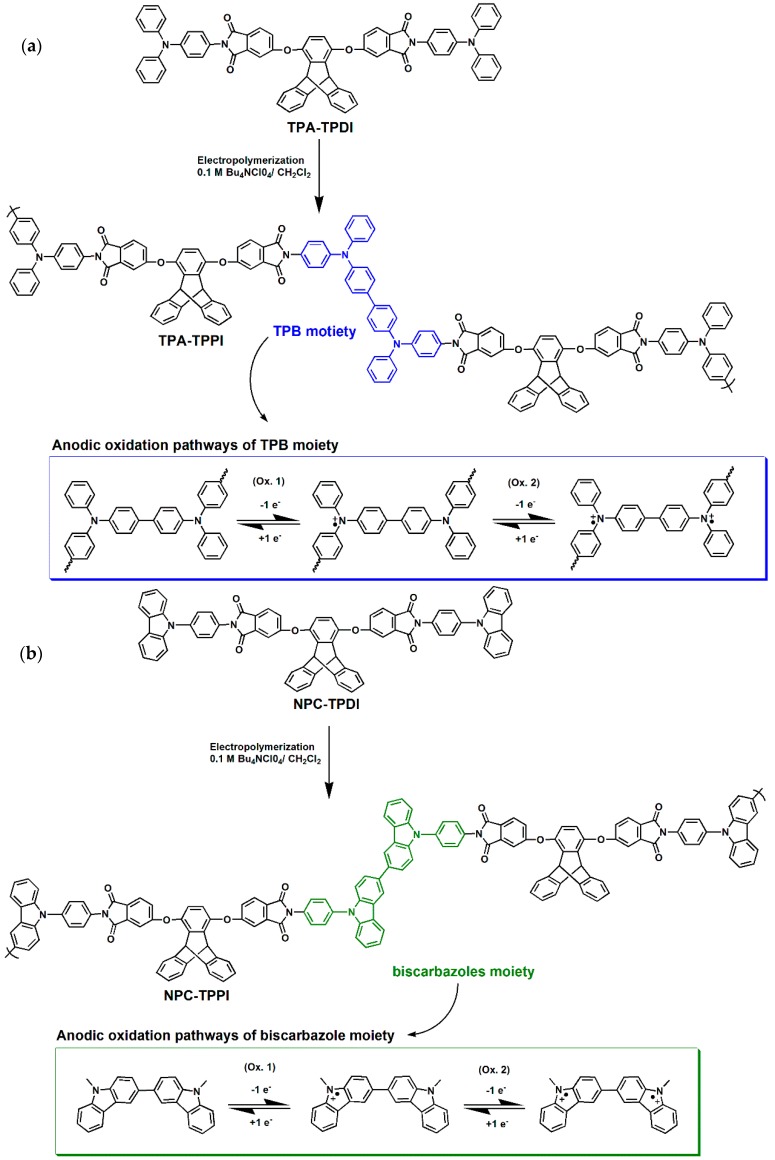
Proposed structures of electro-generated polymers (**a**) TPA–TPPI and (**b**) NPC–TPPI.

The electrochemical behavior of the electrodeposited polymer films was investigated by cyclic voltammetry experiments. The redox cycles of the film samples were measured in dichloromethane using Bu_4_NClO_4_ as the electrolyte. Figure 14 displays the CV diagrams of electrodeposited polymer films of TPA–TPPI and NPC–TPPI on the ITO-coated glass slide in 0.1 M Bu_4_NClO_4_/CH_2_Cl_2_ at a scan rate of 50 mV s^−1^. The TPA–TPPI film shows two redox couples. The first anodic peak corresponds to one-electron oxidations of the TPB units to form radical cations, followed subsequently by the oxidation to dicationic species (Scheme 5a). The NPC–TPPI film also exhibits two reversible redox couples attributed to the radical cation and dication states of biscarbazole, respectively (Scheme 5b). The quantitative details are summarized in Table 1. The half-wave potential (*E*_1/2_) of the first oxidation process of TPA–TPPI and NPC–TPPI were recorded at 0.87 V and 1.01 V (vs. Ag/AgCl), respectively. Oxidation potentials of the polymers calibrated to the ferrocene redox couple (+0.44 V vs. Ag/AgCl) were used for the calculation of HOMO-LUMO energy levels. The HOMO energy levels for TPA–TPPI and NPC–TPPI were calculated to be 5.05 and 5.20 eV (relative to the zero vacuum level), respectively, assuming that the HOMO energy level for the ferrocene/ferrocenium (Fc/Fc^+^) standard is 4.80 eV with respect to the zero vacuum level. After deducing from the bandgap calculated from the UV–Vis absorption onsets, their LUMO energy levels were calculated to be 2.00 and 1.78 eV, respectively. 

**Figure 15:**
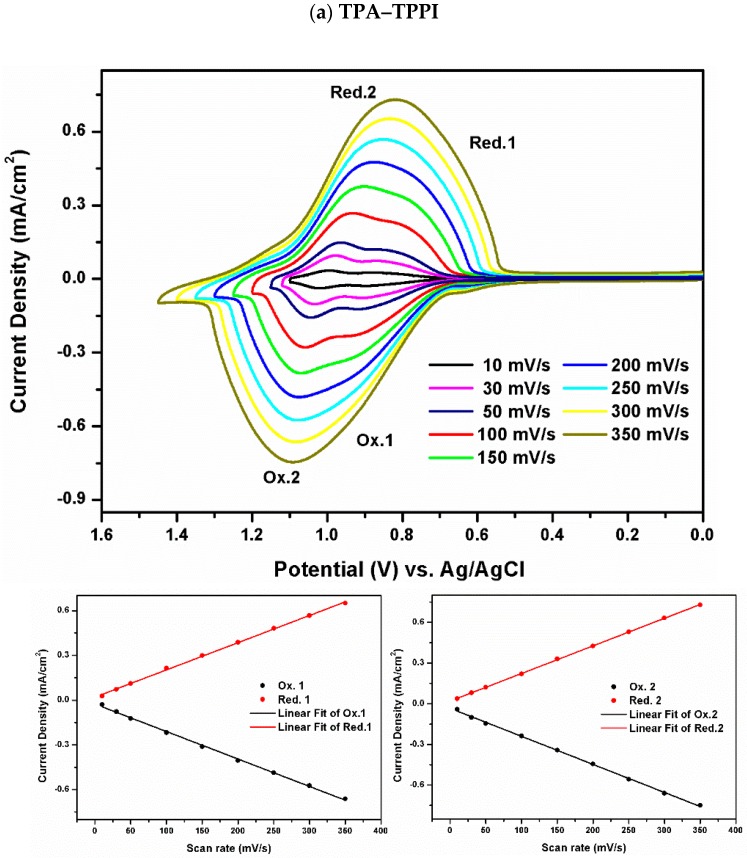

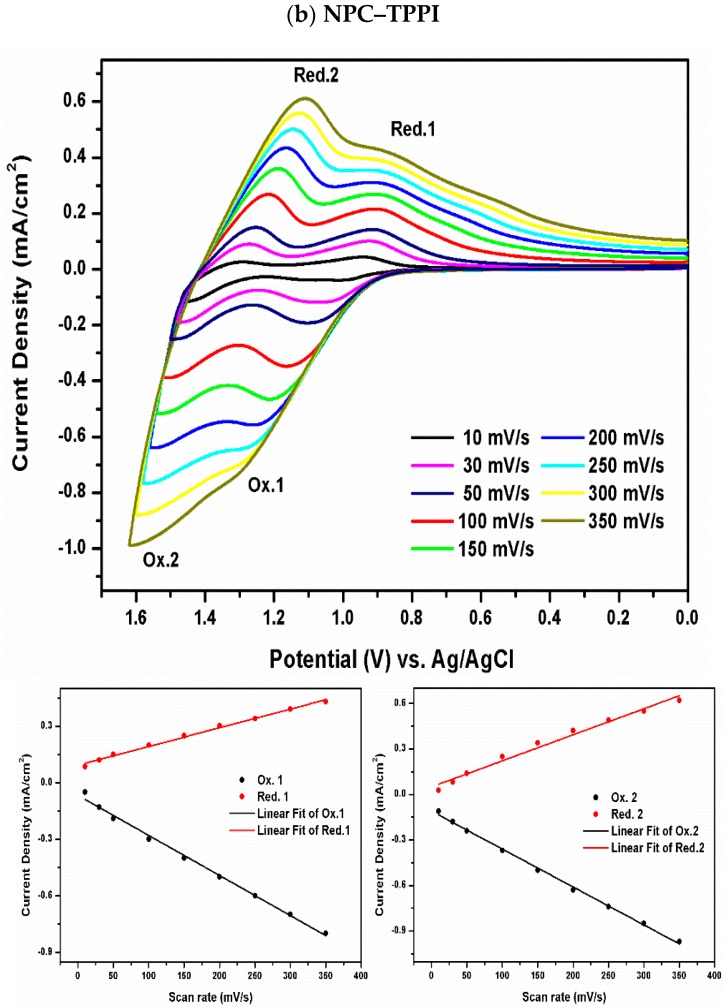
Scan rate dependence of (**a**) TPA–TPPI and (**b**) NPC–TPPI films on the ITO-coated glass slide in CH_2_Cl_2_ containing 0.1 M Bu_4_NClO_4_ at different scan rates between 10 and 350 mV s^−1^.

Figure 15a,b displays the CV curves of the TPA–TPPI and NPC–TPPI at different scanning rates between 10 and 350 mV/s in 0.1 M Bu_4_NClO_4_/CH_2_Cl_2_ solution. The anodic and cathodic peak currents show a linear dependence as a function of the scan rate. This indicates that the electrochemical processes are not diffusion limited and are reversible even at very high scan rates.

### 3.5. Electro-Optical Properties

**Figure 16:**
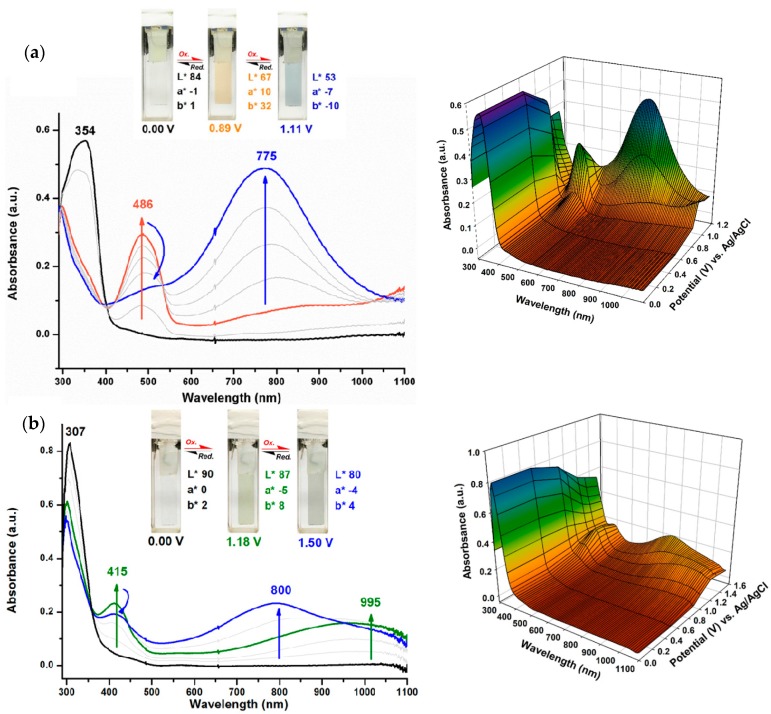
Spectroelectrograms and color changes of (**a**) TPA–TPPI and (**b**) NPC–TPPI thin films on ITO-coated glass in 0.1 M Bu_4_NClO_4_/CH_2_Cl_2_ at various applied voltages.

The electro-optical properties of the electro-generated polymer films were investigated using the changes in electronic absorption spectra at various applied voltages. Spectroelectrochemical series of TPA–TPPI and NPC–TPPI polymer films deposited on ITO were recorded in the monomer-free electrolyte solution. The result of TPA–TPPI film upon electro-oxidation is presented in Figure 16a as a series of UV–Vis–NIR absorption curves correlated to electrode potentials. Before electrical potential was applied, the TPA–TPPI film showed a strong absorption at 354 nm, characteristic for triarylamine π–π* transitions, but it was almost colorless in the visible region. By setting the potential to 0.89 V, the intensity of the absorption band around 354 nm decreased gradually, and the growth of a new peak at 486 nm was observed, accompanied with a yellow-orange color change of the film. As the potential increased more positively to 1.11 V, the absorbance at 486 nm slightly decreased and the broad absorption centered at 775 nm grew up. Meanwhile, the film changed color from yellow-orange to blue. This can be attributed to the successive formation of TPB radical cations and dications, respectively, as shown in Scheme 5a. The film showed a multicolored electrochromism from colorless neutral state to yellow-orange semi-oxidized state and blue fully oxidized state.

Figure 16b shows the spectroelectrograms and color changes of the NPC–TPPI film while incrementally increasing the applied potential between 0 and 1.50 V. In the neutral state, the NPC–TPPI film showed a single strong absorption peak at 307 nm, which corresponded to the π–π* transition. Upon increase of the applied potential to 1.18 V, the absorption at 307 nm decreased, and new peak at 415 nm and a broadband absorption extended to the near-IR (NIR) range appeared, due to charge carrier band formations. The absorption band in NIR region may be attributed to the inter-valance charge transfer (IVCT) effect between states in which the positive charge is centered at different amino center (biscarbazole). It has been demonstrated that the radical cation species of bistriarylamines with delocalized redox centers generally showed strong IVCT absorption bands [43]. Upon further increasing the applied potential to 1.50 V, the dication (bipolaron) band at 800 nm appeared, and the absorption at 415 nm decreased slightly in intensity. The NPC–TPPI film showed color changes upon oxidation, from a colorless neutral state to pale green or blue-green oxidized states. In order to measure these colors in an accurate manner, we performed colorimetry analysis and provided the relevant data in Figure 16.

**Figure 17:**
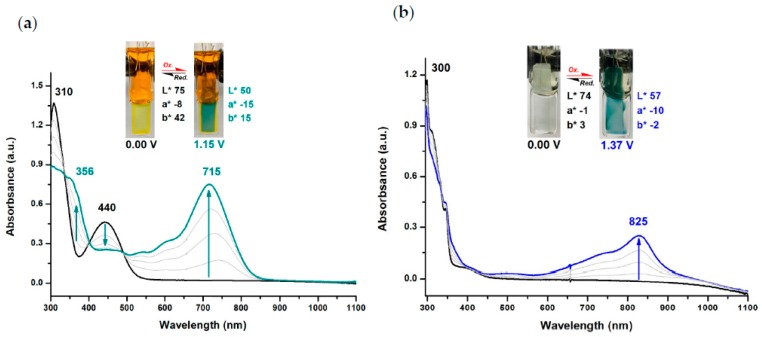
Spectroelectrograms and color changes of 2 mM of (**a**) M1 and (**b**) M2 in 0.1 M Bu_4_NClO_4_/CH_2_Cl_2_ solution at various applied voltages.

We also investigated the electro-optical properties of model compounds at various applied voltages using an OTTLE cell. Figure 17a shows the spectral and color change of 2 mM of M1 upon incremental oxidative scans from 0 to 1.15 V. Before electrical potential was applied, M1 solution showed two absorption bands at 310 and 440 nm. The solution presented yellow color in neutral state possibly because of the intramolecular charge transfer (CT) interactions between alternating electron-donating and electron-withdrawing groups. By adjusting the potential to 1.15 V, the intensity of the absorption bands 310 and 440 nm decreased gradually, and the growth of two new peaks at 356 and 715 nm were observed. Meanwhile, the color of ITO changes into cyan. The spectral and color change are believed to be related the oxidation of the TPA unit. As seen in Figure 17b, significant change in the absorption spectrum of M2 occurred upon applying a potential of 1.37 V. The resulting oxidized form was steel blue and absorbed at 825 nm while the neutral form was almost colorless and absorbed at 300 nm. The spectral and color changes are believed to be related the oxidation of the carbazole unit.

### 3.6. Electrochromic Switching

**Figure 18:**
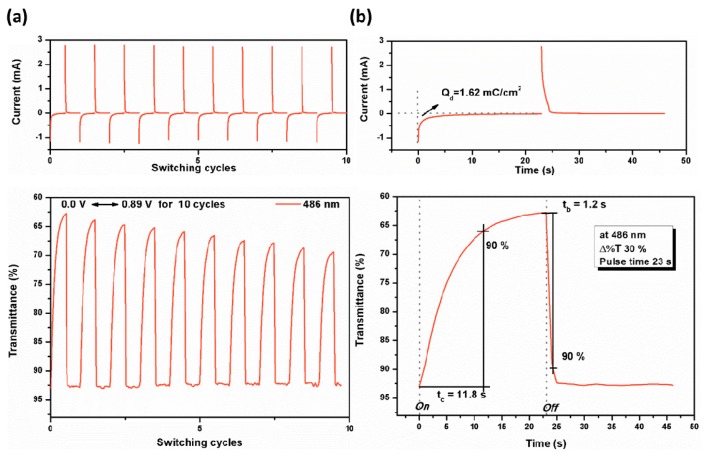
Potential step absorptiometry of the cast film of TPA–TPPI on the ITO-glass slide (coated area ~1 cm^2^) (in CH_2_Cl_2_ with 0.1 M Bu_4_NClO_4_ as the supporting electrolyte) by appling a potential step; (**a**) optical switching at potential 0.00 V ⬄ 0.89 V (10 cycles) with a pulse width of 23 s, monitored at λ_max_ = 486 nm; (**b**) the 1st cycle transmitance change for the TPA–TPPI thin film.

**Figure 19:**
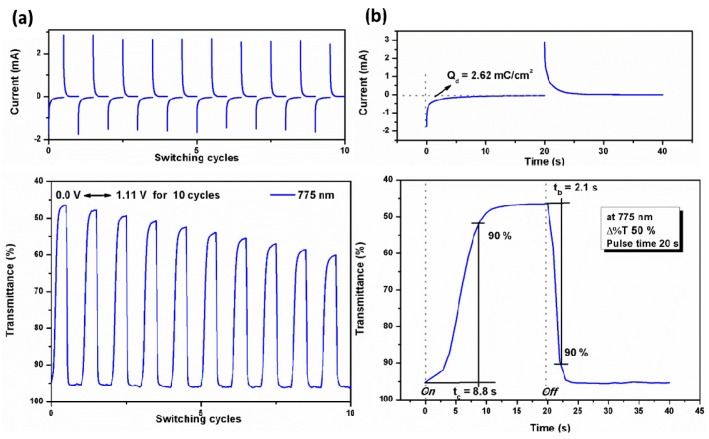
Potential step absorptiometry of the cast film of TPA–TPPI on the ITO-glass slide (coated area ~1 cm^2^) (in CH_2_Cl_2_ with 0.1 M Bu_4_NClO_4_ as the supporting electrolyte) by appling a potential step; (**a**) optical switching at potential 0.00 V ⬄ 1.11 V (10 cycles) with a pulse width of 20 s, monitored at λ_max_ = 775 nm; (**b**) the 1st cycle transmitance change for the TPA–TPPI thin film.

Electrochromic switching studies for the electrodeposited polymer films were performed to monitor the percent transmittance changes (Δ%*T*) as a function of time at their absorption maximum and to determine the response time by stepping potential repeatedly between their neutral and oxidized states. The active area of the polymer film on ITO glass is about 1 cm^2^. While the films were switched, the absorbance at selected wavelengths was monitored as a function of time with UV–Vis–NIR spectroscopy. Figure 18 and Figure 19 depict the optical transmittance of TPA–TPPI film at 486 and 775 nm as a function of time by applying square-wave potential steps between 0 and 0.89 V for a pulse time of 23 s and between 0 and 1.11 V for a pulse time of 20 s. The response time was calculated at 90% of the full-transmittance change, because it is difficult to perceive any further color change with naked eye beyond this point. As shown in Figure 14b, TPA–TPPI attained 90% of a complete coloring and bleaching in 11.8 and 1.2 s, respectively. The optical contrast measured as Δ%*T* of TPA–TPPI between neutral colorless and oxidized yellow-orange states was found to be 30% at 486 nm. Then the applied voltage was stepped between 0 and 1.11 V, the TPA–TPPI exhibited Δ%*T* up to 50% at 775 nm for oxidized blue states and required 8.8 s for the coloring process and 2.1 s for the bleaching process (Figure 19b). In addition, the electrochromic coloration efficiency (CE; η) can be calculated via optical density using the equation (η = ΔOD/Q_d_) where ΔOD is the optical absorbance change, and *Q*_d_ (mC cm^−2^) is the inject/ejected charge during a redox step. On the basis of this equation, the CE values of TPA–TPPI estimated from the data shown in Table 2 were found to be 105 cm^2^ C^−1^ at 486 nm and 118 cm^2^ C^−1^ at 775 nm.

The polymer film of NPC–TPPI was switched between 0 and 1.18 V for a pulse width of 16 s. The results are shown in Figure S7 (Supplementary Materials). In this case, a response time required for 90% full-transmittance change of 6.7 s for the coloration step and 5.0 s for the bleaching step at 415 nm. In addition, the optical contrast measured as Δ%*T* recorded at neutral and oxidized form was found to be 30%. The CE value of NPC–TPPI was calculated as 78 cm^2^ C^−1^.

### 3.7. Electrochromic Devices

**Figure 20:**
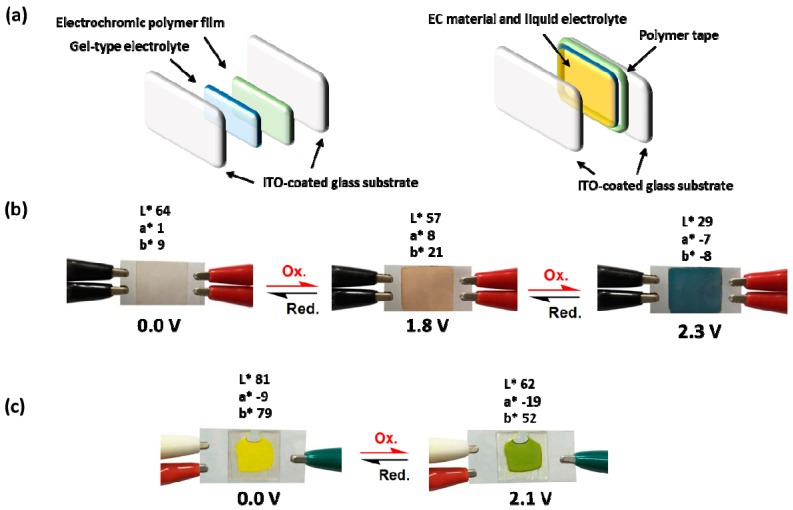
(**a**) Schematic illustration of the structure of the electrochromic device; (**b**) Image of the sandwich-type ITO-coated glass electrochromic cell, using TPA–TPPI as active layer; (**c**) Image of the liquid type ECD using the Bu_4_NClO_4_/propylene carbonate solution of M1 as the electrochromic layer.

Finally, we fabricated single layer electrochromic cells (Figure 20) as preliminary investigations for the electrochromic applications of the electrodeposited polymers and model compounds. As a representative example, the polymer film TPA–TPPI was electrodeposited onto ITO-coated glass, thoroughly rinsed, and then dried. Afterwards, the gel electrolyte was spread on the polymer-coated side of the electrode and the electrodes were sandwiched as shown in Figure 20b. The TPA–TPPI film is colorless in the neutral form. When the applied voltage was increased to 1.8 V, the color changed to a yellow-orange. Upon further oxidation at applied potential to 2.3 V, the color changed to blue. When the potential was subsequently set back at 0.0 V, the polymer film gradually turned back to original color. The liquid type ECD based on the Bu_4_NClO_4_/propylene carbonate solution of M1 could switch cyan color change at applied voltage of 2.1 V (Figure 20c).

## 4. Conclusions

Two new bisimide monomers TPA–TPDI and NPC–TPDI with the triptycene unit as an interior core and triphenylamine or *N*-phenylcarbazole end groups were successfully synthesized and characterized. For a comparative study, two model compounds M1 and M2 with *tert*-butyl groups blocked on the reactive sites of their terminal TPA and NPC units were also prepared and characterized for electrochemical and electrochromic properties. Polymer films could be built on the electrode surface from TPA–TPDI and NPC–TPDI in electrolyte solutions by repetitive CV scanning. The electro-deposited films exhibit reversible electrochemical oxidation accompanied by color changes having moderate coloration efficiency. The color of TPA–TPPI and NPC–TPPI films change from the almost colorless neutral state to yellow-orange and blue oxidized states when scanning from 0.00 to 1.11 V, and from colorless neutral form to pale green and pale blue-green oxidized forms when scanning from 0.00 to 1.50 V, respectively. In contrast, model compounds M1 and M2 showed reversible oxidation processes during repeated CV scanning and exhibited obvious electrochromic behavior nearby the ITO electrode surface in an OTTLE cell. These characteristics suggest that the electro-deposited polymers and the model compounds could be good candidates to act as anodically electrochromic materials. 

## Figures and Tables

**Table 1:** Optical and electrochemical properties of the electro-synthesized polymers.

Polymer	UV–Vis absorption (nm) ^a^		Oxidation potential (V) ^b^			*E*_g_ (eV) ^c^	HOMO (eV) ^d^	LUMO (eV) ^d^
	λ_max_	λ_onset_	*E*_onest_	*E*_1/2_^Ox1^	*E*_1/2_^Ox2^			
TPA–TPPI	354	407	0.69	0.87	1.00	3.05	5.05	2.00
NPC–TPPI	307	363	0.84	1.01	1.36	3.42	5.20	1.78

Slide in 0.1 M Bu_4_NClO_4_/CH_2_Cl_2_ at a scan rate of 50 mV s^−1^. ^a^ UV–Vis absorption maximum and onset for the polymer films; ^b^ Read from first CV scans, versus Ag/AgCl in CH_2_Cl_2_ at a scan rate of 50 mV s^−1^; ^c^ Optical bandgap calculated from absorption edge of the polymer film: *E*_g_ = 1240/λ_onset_; ^d^ The HOMO and LUMO energy levels were calculated from *E*_onest_ values of CV diagrams and were referenced to ferrocene (4.8 eV relative to vacuum energy level; *E*_onest_ = 0.44 V in CH_2_Cl_2_); *E*_HOMO_ = *E*_onest_ + 4.8 − 0.44 (eV); *E*_LUMO_ = *E*_HOMO_ − *E*_g_.

**Table 2:** Electrochromic properties of the polymer films.

Polymer	λ_max_^a^ (nm)	Δ%*T*	Response time ^b^		ΔOD ^c^	*Q*_d_^d^ (mC cm^−2^)	CE ^e^ (cm^2^ C^−1^)
			*t*_c_ (s)	*t*_b_ (s)			
TPA-TPPI	486	30	11.8	1.2	0.17	1.62	105
	775	50	8.8	2.1	0.31	2.62	118
NPC-TPPI	415	30	6.7	5.0	0.21	2.68	78

^a^ Wavelength of absorption maximum; ^b^ Time for 90% of the full-transmittance change; ^c^ Optical Density (ΔOD) = log(*T*_bleached_/*T*_colored_), where *T*_colored_ and *T*_bleached_ are the maximum transmittance in the oxidized and neutral states, respectively; ^d^ *Q*_d_ is ejected charge, determined from the in situ experiments; ^e^ Coloration efficiency (CE) = ΔOD/*Q*_d_.

## Supplementary Materials

The following are available online at www.mdpi.com/2073-4360/9/10/497/s1, Figure S1: IR spectra of model compounds M1 and M2, together with compounds **4**, **7** and **8**; Figure S2: (a) ^1^H NMR and (b) H-H COSY spectra of M1 in DMSO-*d*6; Figure S3: (a) ^1^H NMR and (b) H-H COSY spectra of M2 in CDCl_3_; Figure S4−S6: Mass spectra of the synthesized compounds; Figure S7: Potential step absorptiometry of the cast film of NPC-TPPI on the ITO-glass slide (coated area ~1 cm^2^) (in CH_2_Cl_2_ with 0.1 M Bu_4_NClO_4_ as the supporting electrolyte) by applying a potential step.
