# Supplementary material for: Facile Synthesis of Electroactive and Electrochromic Triptycene Poly(ether-imide)s Containing Triarylamine Units via Oxidative Electro-Coupling
Source: Polymers (Basel). 2017 Oct 10;9(10):497. doi: 10.3390/polym9100497 (PMC6418647; doi:10.3390/polym9100497)
Supplement: Supplementary file 1 [file polymers-09-00497-s001.pdf]

Supplementary Materials

Facile Synthesis of Electroactive and Electrochromic Triptycene Poly(ether-imide)s Containing Triarylamine Units via Oxidative Electro-coupling

Sheng-Huei Hsiao*, Yu-Chuan Liao

Department of Chemical Engineering and Biotechnology, National Taipei University of Technology, No. 1, Sec. 3, Chunghsiao East Rd., Taipei 10608, Taiwan

* Correspondence: shhsiao@ntut.edu.tw; Tel.: +886-2-27712171 ext. 2548; Fax: +886-2-27317117

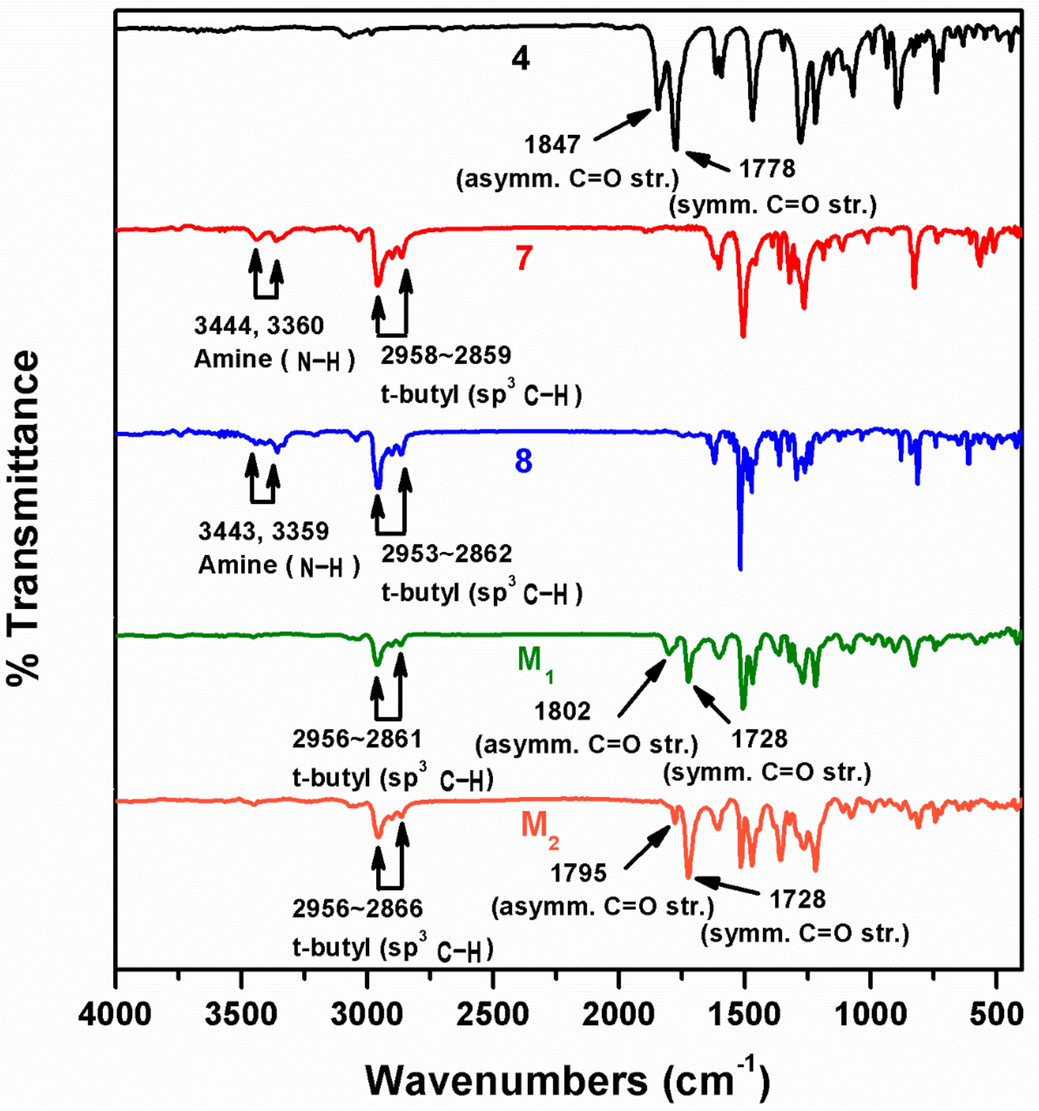

Figure S1. IR spectra of model compounds M_1 and M_2, together with compounds **4**, **7** and **8**.

(a)

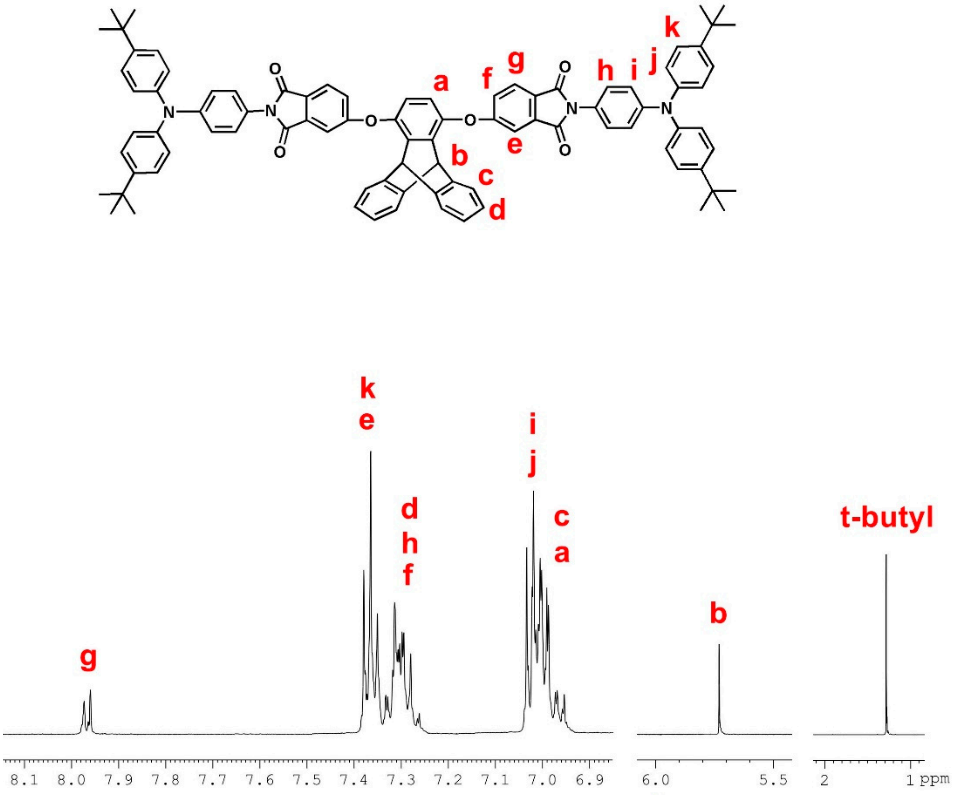

(b)

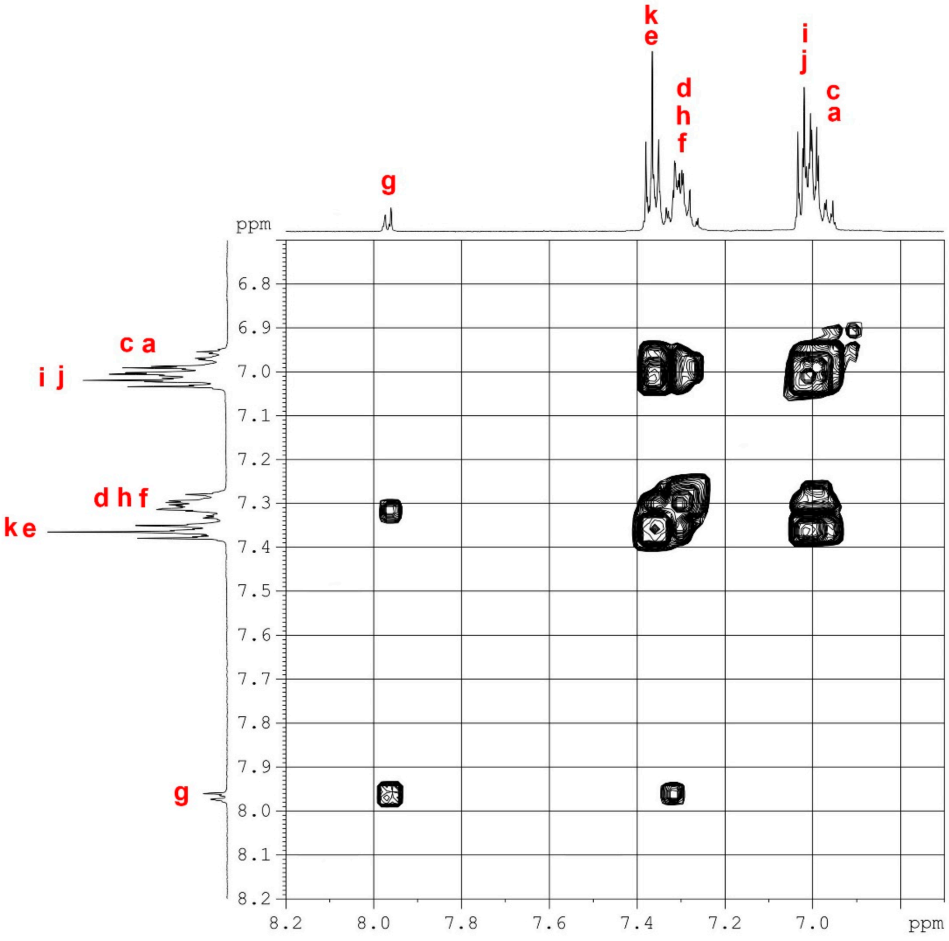

Figure S2. (a) ^{1}H NMR and (b) H-H COSY spectra of $\mathbf{M_1}$ in DMSO-d_6.

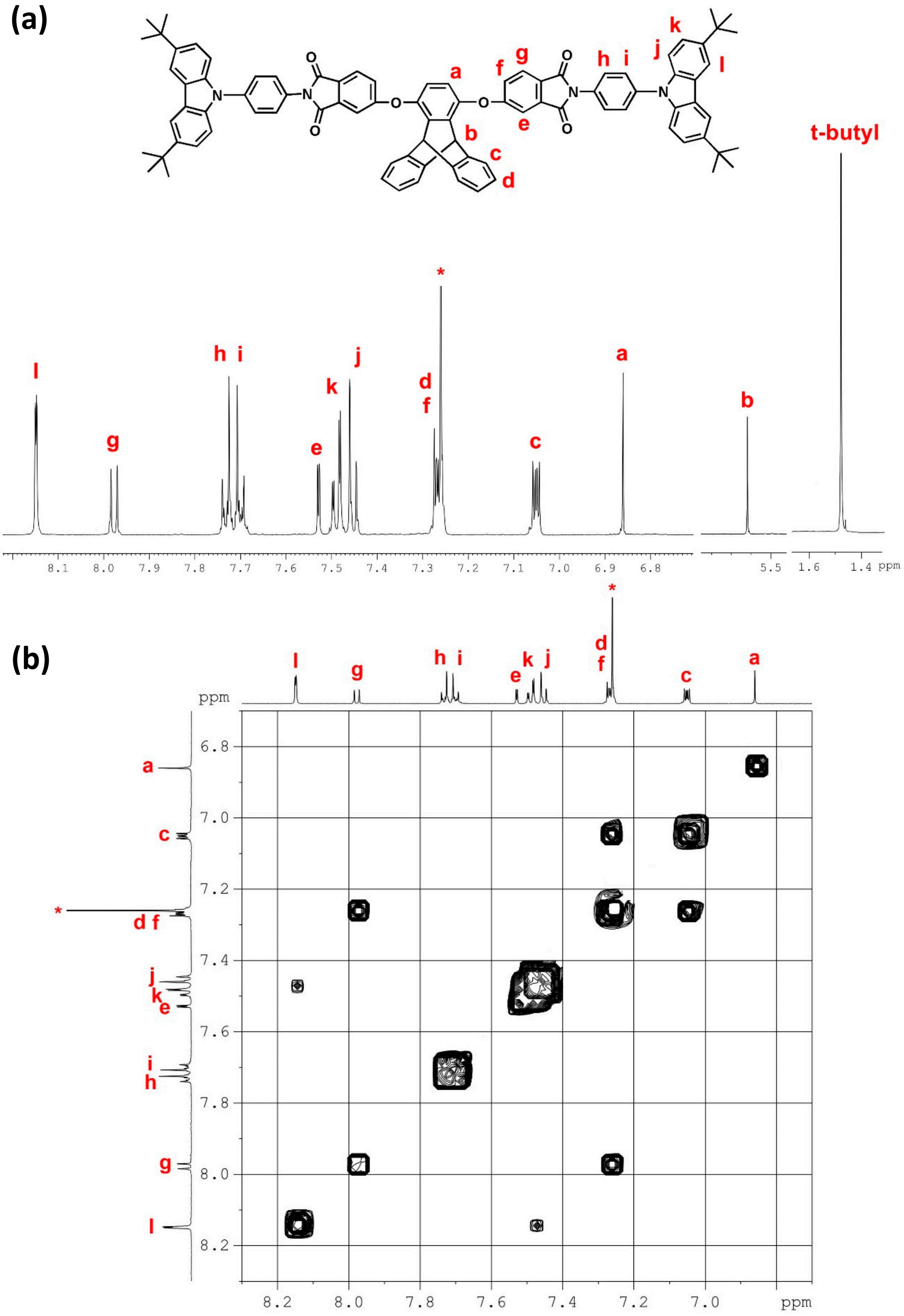

Figure S3. (a) ^{1}H NMR and (b) H-H COSY spectra of **M$_2$** in $CDCl_3$ (* solvent peak).

Elemental Composition Report **Page 1 of 1**

Single Mass Analysis
Tolerance = 200.0 PPM / DBE: min = -1.5, max = 100.0
Element prediction: Off
Number of isotope peaks used for i-FIT = 3

Monoisotopic Mass, Even Electron Ions
172 formula(e) evaluated with 41 results within limits (all results (up to 1000) for each mass)
Elements Used:
C: 70-80 H: 40-100 N: 1-5 O: 1-10 Na: 1-1
TPA
170421esi06 592 (5.746) Cm (592-(569+616))

21-Apr-2017
1: TOF MS ES+
2.88e+005

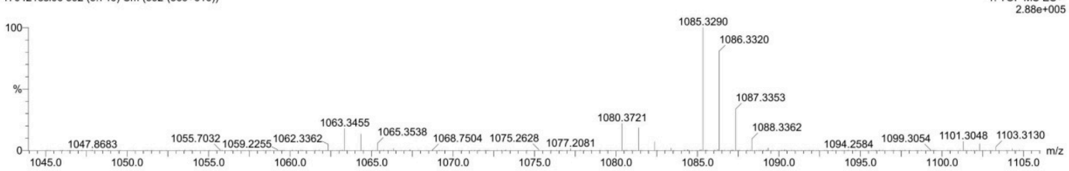

		mDa	PPM	DBE	
Minimum:				-1.5	
Maximum:		5.0	200.0	100.0	

Mass	Calc. Mass	mDa	PPM	DBE	Formula
1085.3290	1085.3315	-2.5	-2.3	51.5	C72 H46 N4 O6 Na
	1085.3256	3.4	3.1	60.5	C79 H42 N4 O Na
	1085.3355	-6.5	-6.0	55.5	C77 H46 N2 O4 Na
	1085.3203	8.7	8.0	51.5	C73 H46 N2 O7 Na
	1085.3414	-12.4	-11.4	46.5	C70 H50 N2 O9 Na
	1085.3144	14.6	13.5	60.5	C80 H42 N2 O2 Na
	1085.3468	-17.8	-16.4	55.5	C76 H46 N4 O3 Na
	1085.3104	18.6	17.1	56.5	C75 H42 N4 O4 Na
	1085.3567	-27.7	-25.5	50.5	C74 H50 N2 O6 Na
	1085.2991	29.9	27.5	56.5	C76 H42 N2 O5 Na
	1085.2951	33.9	31.2	52.5	C71 H42 N4 O7 Na
	1085.3679	-38.9	-35.8	50.5	C73 H50 N4 O5 Na
	1085.3719	-42.9	-39.5	54.5	C78 H50 N2 O3 Na
	1085.2839	45.1	41.6	52.5	C72 H42 N2 O8 Na
	1085.3778	-48.8	-45.0	45.5	C71 H54 N2 O8 Na
	1085.3831	-54.1	-49.8	54.5	C77 H50 N4 O2 Na
	1085.3890	-60.0	-55.3	45.5	C70 H54 N4 O7 Na
	1085.3930	-64.0	-59.0	49.5	C75 H54 N2 O5 Na
	1085.4043	-75.3	-69.4	49.5	C74 H54 N4 O4 Na
	1085.4083	-79.3	-73.1	53.5	C79 H54 N2 O2 Na
	1085.4142	-85.2	-78.5	44.5	C72 H58 N2 O7 Na
	1085.4195	-90.5	-83.4	53.5	C78 H54 N4 O Na
	1085.4254	-96.4	-88.8	44.5	C71 H58 N4 O6 Na
	1085.4294	-100.4	-92.5	48.5	C76 H58 N2 O4 Na
	1085.4407	-111.7	-102.9	48.5	C75 H58 N4 O3 Na
	1085.4447	-115.7	-106.6	52.5	C80 H58 N2 O Na
	1085.4506	-121.6	-112.0	43.5	C73 H62 N2 O6 Na

Sample name	分子式	結構式	Exact mass
TPA	$C_{72}H_{46}N_4O_6$	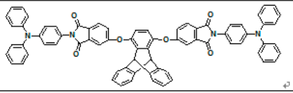	1062.34

Elemental Composition Report **Page 1 of 1**

Single Mass Analysis
Tolerance = 200.0 PPM / DBE: min = -1.5, max = 100.0
Element prediction: Off
Number of isotope peaks used for i-FIT = 3

Monoisotopic Mass, Even Electron Ions
166 formula(e) evaluated with 35 results within limits (all results (up to 1000) for each mass)
Elements Used:
C: 70-80 H: 40-100 N: 1-5 O: 1-10 Na: 1-1
NPC
170421esi07 588 (5.712) Cm (588-(576+597))

21-Apr-2017
1: TOF MS ES+
1.47e+004

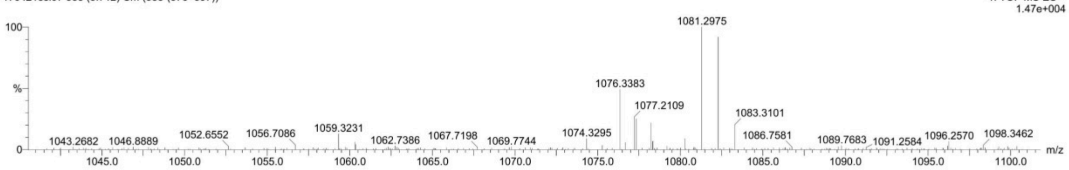

		mDa	PPM	DBE	
Minimum:				-1.5	
Maximum:		5.0	200.0	100.0	

Mass	Calc. Mass	mDa	PPM	DBE	Formula
1081.2975	1081.3002	-2.7	-2.5	53.5	C72 H42 N4 O6 Na
	1081.3042	-6.7	-6.2	57.5	C77 H42 N2 O4 Na
	1081.2890	8.5	7.9	53.5	C73 H42 N2 O7 Na
	1081.3101	-12.6	-11.7	48.5	C70 H46 N2 O9 Na
	1081.3155	-18.0	-16.6	57.5	C76 H42 N4 O3 Na
	1081.3254	-27.9	-25.8	52.5	C74 H46 N2 O6 Na
	1081.3366	-39.1	-36.2	52.5	C73 H46 N4 O5 Na
	1081.3406	-43.1	-39.9	56.5	C78 H46 N2 O3 Na
	1081.3465	-49.0	-45.3	47.5	C71 H50 N2 O8 Na
	1081.3518	-54.3	-50.2	56.5	C77 H46 N4 O2 Na
	1081.3577	-60.2	-55.7	47.5	C70 H50 N4 O7 Na
	1081.3617	-64.2	-59.4	51.5	C75 H50 N2 O5 Na
	1081.3730	-75.5	-69.8	51.5	C74 H50 N4 O4 Na
	1081.3770	-79.5	-73.5	55.5	C79 H50 N2 O2 Na
	1081.3829	-85.4	-79.0	46.5	C72 H54 N2 O7 Na
	1081.3882	-90.7	-83.9	55.5	C78 H50 N4 O Na
	1081.3941	-96.6	-89.3	46.5	C71 H54 N4 O6 Na
	1081.3981	-100.6	-93.0	50.5	C76 H54 N2 O4 Na
	1081.4094	-111.9	-103.5	50.5	C75 H54 N4 O3 Na
	1081.4134	-115.9	-107.2	54.5	C80 H54 N2 O Na
	1081.4193	-121.8	-112.6	45.5	C73 H58 N2 O6 Na
	1081.4305	-133.0	-123.0	45.5	C72 H58 N4 O5 Na
	1081.4345	-137.0	-126.7	49.5	C77 H58 N2 O3 Na
	1081.4404	-142.9	-132.2	40.5	C70 H62 N2 O8 Na
	1081.4457	-148.2	-137.1	49.5	C76 H58 N4 O2 Na
	1081.4556	-158.1	-146.2	44.5	C74 H62 N2 O5 Na
	1081.4669	-169.4	-156.7	44.5	C73 H62 N4 O4 Na

Sample name	分子式	結構式	Exact mass
NPC	$C_{72}H_{42}N_4O_6$	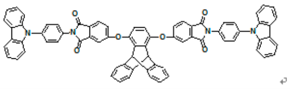	1058.31

Figure S4. Mass spectra of monomers **TPA-TPDI** and **NPC-TPDI.**

Sample name	分子式	結構式	Exact mass
TPA-tBu	$C_{88}H_{78}N_4O_6$	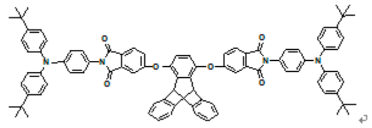	1286.59

[Mass Spectrum]
Data : 20170425fab(+)_011_TPA-tBu Date : 25-Apr-2017 15:15
RT : 3.00 min Scan# : (106,115)
Elements : C 100/0, H 100/0, N 4/0, O 6/0
Mass Tolerance : 100ppm, 5mmu if m/z < 50, 50mmu if m/z > 500
Unsaturation (U.S.) : -0.5 – 100.0

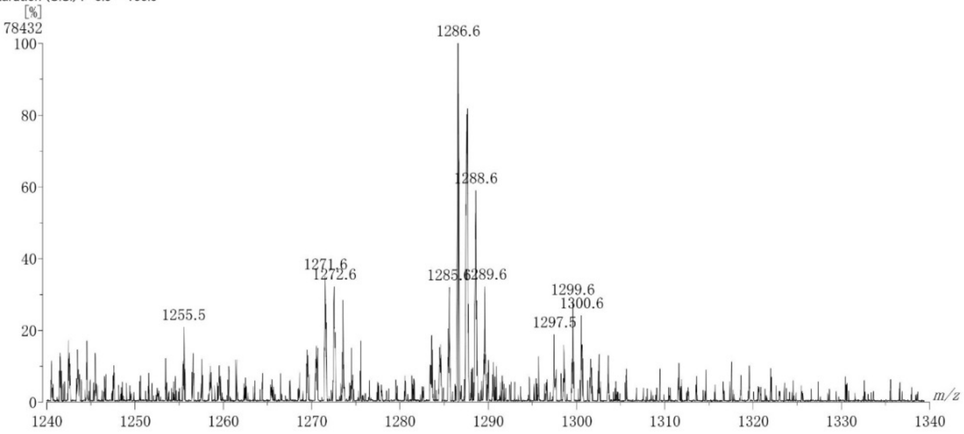

	Observed m/z	Int%	Err[ppm / mmu]	U.S.	Composition
1	1286.5910	100.00	+19.1 / +24.5	65.5	C100 H72 N
2			+28.8 / +37.1	66.0	C99 H70 N2
3			+38.6 / +49.7	66.5	C98 H68 N3
4			-34.4 / -44.2	59.5	C97 H80 N3
5			-24.6 / -31.6	60.0	C96 H78 N4
6			+37.6 / +48.3	66.0	C100 H70 O
7			-35.4 / -45.6	59.0	C99 H82 O
8			-25.6 / -33.0	59.5	C98 H80 N O
9			-15.9 / -20.4	60.0	C97 H78 N2 O
10			-6.1 / -7.8	60.5	C96 H76 N3 O
11			+3.7 / +4.7	61.0	C95 H74 N4 O
12			-7.1 / -9.2	60.0	C98 H78 O2
13			+2.6 / +3.4	60.5	C97 H76 N O2
14			+12.4 / +16.0	61.0	C96 H74 N2 O2
15			+22.2 / +28.5	61.5	C95 H72 N3 O2
16			+32.0 / +41.1	62.0	C94 H70 N4 O2
17			+21.1 / +27.2	61.0	C97 H74 O3
18			+30.9 / +39.8	61.5	C96 H72 N O3
19			-32.3 / -41.5	55.0	C94 H82 N2 O3
20			-22.5 / -29.0	55.5	C93 H80 N3 O3
21			-12.7 / -16.4	56.0	C92 H78 N4 O3
22			-23.6 / -30.3	55.0	C95 H82 O4
23			-13.8 / -17.7	55.5	C94 H80 N O4
24			-4.0 / -5.2	56.0	C93 H78 N2 O4
25			+5.8 / +7.4	56.5	C92 H76 N3 O4
26			+15.5 / +20.0	57.0	C91 H74 N4 O4
27			+4.7 / +6.1	56.0	C94 H78 O5
28			+14.5 / +18.6	56.5	C93 H76 N O5
29			+24.3 / +31.2	57.0	C92 H74 N2 O5
30			+34.0 / +43.8	57.5	C91 H72 N3 O5
31			-29.2 / -37.5	51.0	C89 H82 N4 O5
32			+33.0 / +42.5	57.0	C93 H74 O6
33			-30.2 / -38.9	50.5	C91 H84 N O6
34			-20.4 / -26.3	51.0	C90 H82 N2 O6
35			-10.7 / -13.7	51.5	C89 H80 N3 O6
36			-0.9 / -1.1	52.0	C88 H78 N4 O6

Figure S5. Mass spectra of model compound **M1**.

Sample name	分子式	結構式	Exact mass
NPC-tBu	$C_{88}H_{74}N_4O_6$	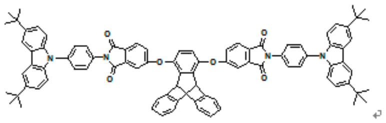	1282.56

[Mass Spectrum]
Data : 20170425fab(+)_013_NPC-tBu   Date : 25-Apr-2017 15:43
RT : 3.46 min   Scan# : (122,127)
Elements : C 100/0, H 100/0, N 4/0, O 6/0
Mass Tolerance   : 30ppm, 5mmu if m/z < 167, 50mmu if m/z > 1667
Unsaturation (U.S.) : -0.5 – 100.0

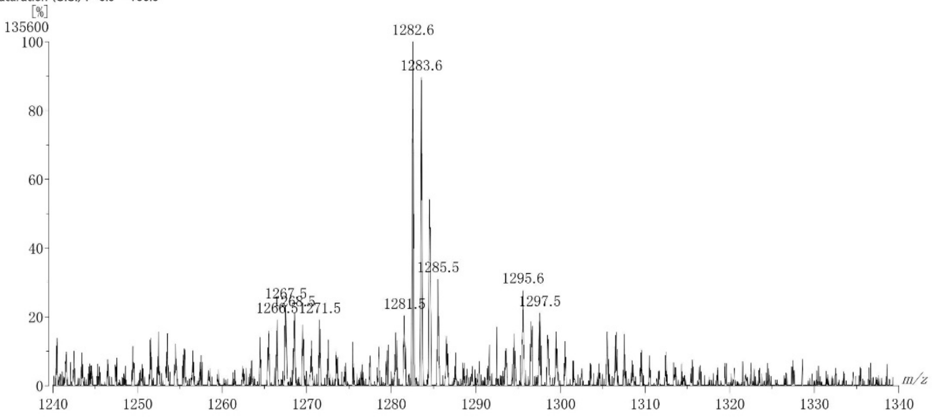

	Observed m/z	Int%	Err[ppm / mmu]	U.S.	Composition
1	1282.5611	100.00	+20.2 / +25.9	67.5	C100 H68 N
2			-23.6 / -30.2	62.0	C96 H74 N4
3			-24.6 / -31.6	61.5	C98 H76 N O
4			-14.8 / -19.0	62.0	C97 H74 N2 O
5			-5.0 / -6.4	62.5	C96 H72 N3 O
6			+4.8 / +6.1	63.0	C95 H70 N4 O
7			-6.1 / -7.8	62.0	C98 H74 O2
8			+3.7 / +4.8	62.5	C97 H72 N O2
9			+13.5 / +17.4	63.0	C96 H70 N2 O2
10			+23.3 / +29.9	63.5	C95 H68 N3 O2
11			+22.3 / +28.6	63.0	C97 H70 O3
12			-21.5 / -27.6	57.5	C93 H76 N3 O3
13			-11.7 / -15.0	58.0	C92 H74 N4 O3
14			-22.5 / -28.9	57.0	C95 H78 O4
15			-12.7 / -16.3	57.5	C94 H76 N O4
16			-2.9 / -3.8	58.0	C93 H74 N2 O4
17			+6.9 / +8.8	58.5	C92 H72 N3 O4
18			+16.7 / +21.4	59.0	C91 H70 N4 O4
19			+5.8 / +7.5	58.0	C94 H74 O5
20			+15.6 / +20.1	58.5	C93 H72 N O5
21			+25.4 / +32.6	59.0	C92 H70 N2 O5
22			-28.2 / -36.1	53.0	C89 H78 N4 O5
23			-29.2 / -37.5	52.5	C91 H80 N O6
24			-19.4 / -24.9	53.0	C90 H78 N2 O6
25			-9.6 / -12.3	53.5	C89 H76 N3 O6
26			+0.2 / +0.3	54.0	C88 H74 N4 O6
27	1283.5663	89.64	+18.2 / +23.3	67.0	C100 H69 N
28			+27.9 / +35.9	67.5	C99 H67 N2
29			-25.6 / -32.9	61.5	C96 H75 N4
30			-26.7 / -34.2	61.0	C98 H77 N O
31			-16.9 / -21.6	61.5	C97 H75 N2 O
32			-7.1 / -9.1	62.0	C96 H73 N3 O
33			+2.7 / +3.5	62.5	C95 H71 N4 O
34			-8.1 / -10.4	61.5	C98 H75 O2
35			+1.7 / +2.2	62.0	C97 H73 N O2
36			+11.5 / +14.7	62.5	C96 H71 N2 O2
37			+21.3 / +27.3	63.0	C95 H69 N3 O2
38			+20.2 / +26.0	62.5	C97 H71 O3
39			-23.5 / -30.2	57.0	C93 H77 N3 O3
40			-13.7 / -17.6	57.5	C92 H75 N4 O3
41			-24.6 / -31.5	56.5	C95 H79 O4
42			-14.8 / -19.0	57.0	C94 H77 N O4
43			-5.0 / -6.4	57.5	C93 H75 N2 O4
44			+4.8 / +6.2	58.0	C92 H73 N3 O4
45			+14.6 / +18.8	58.5	C91 H71 N4 O4
46			+3.8 / +4.8	57.5	C94 H75 O5
47			+13.6 / +17.4	58.0	C93 H73 N O5
48			+23.4 / +30.0	58.5	C92 H71 N2 O5
49			-21.4 / -27.5	52.5	C90 H79 N2 O6
50			-11.6 / -14.9	53.0	C89 H77 N3 O6
51			-1.8 / -2.4	53.5	C88 H75 N4 O6

Figure S6. Mass spectra of model compound **M2**.

(a) **(b)**

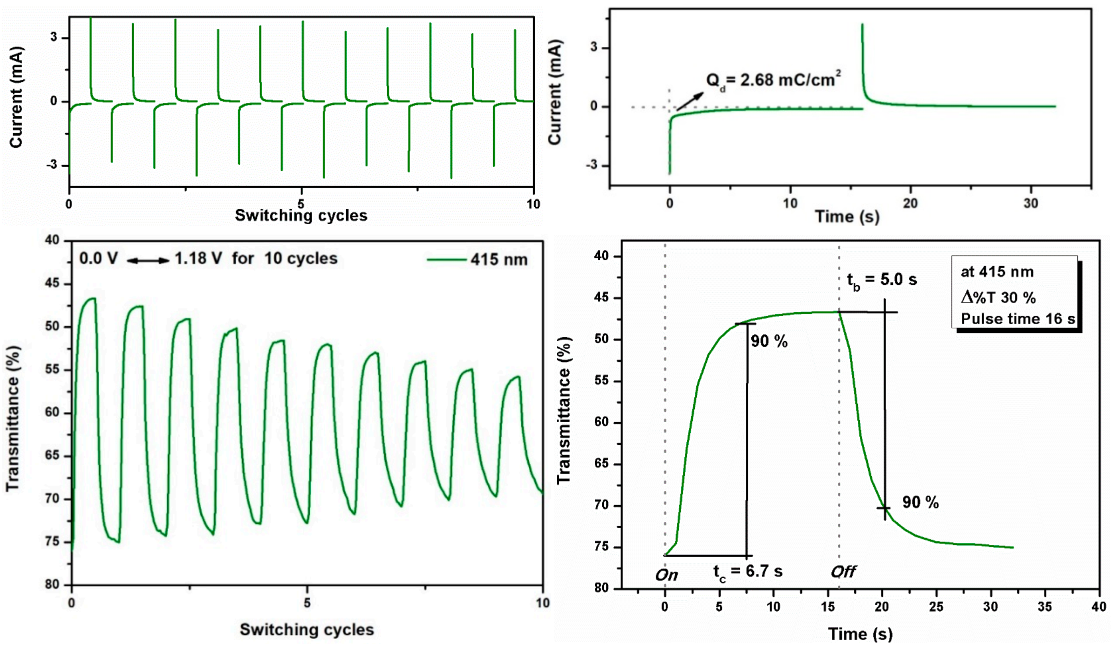

Figure S7. Potential step absorptiometry of the cast film of **NPC-TPPI** on the ITO-glass slide (coated area ~ 1 cm^2) (in CH_2Cl_2 with 0.1 M Bu_4NClO_4 as the supporting electrolyte) by applying a potential step; (a) optical switching at potential 0.00 V ⇔ 1.18 V (10 cycles) with a pulse width of 16 s, monitored at λ_{max} = 415 nm; (b) the 1st cycle transmitance change for the **NPC-TPPI** thin film.
